# Polymeric Microreactors with pH-Controlled Spatial Localization of Cascade Reactions

**DOI:** 10.1021/acsami.3c09196

**Published:** 2023-10-30

**Authors:** Tsvetomir Ivanov, Shoupeng Cao, Nitin Bohra, Marina de Souza Melchiors, Lucas Caire da Silva, Katharina Landfester

**Affiliations:** Department of Physical Chemistry of Polymers, Max Planck Institute for Polymer Research, Ackermannweg 10, 55128 Mainz, Germany

**Keywords:** polymersomes, coacervates, biomimetic membranes, microfluidics, microreactors

## Abstract

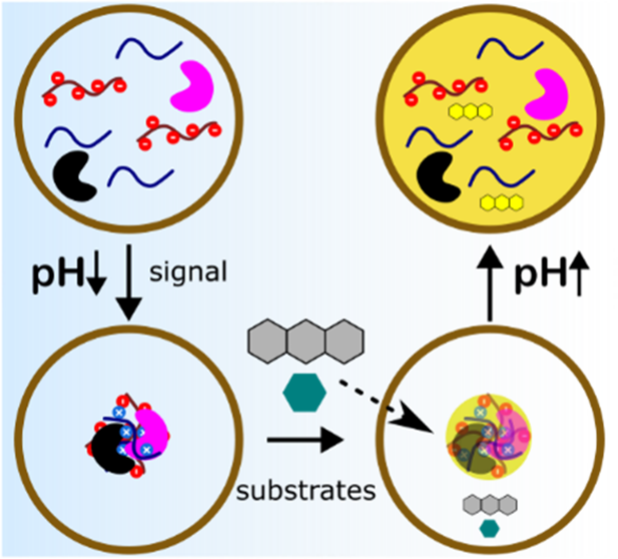

Lipid and polymer vesicles provide versatile means of creating systems that mimic the architecture of cells. However, these constructs cannot mimic the adaptive compartmentalization observed in cells, where the assembly and disassembly of subcompartments are dynamically modulated by environmental cues. Here, we describe a fully polymeric microreactor with a coacervate-in-vesicle architecture that exhibits an adaptive response to pH. The system was fabricated by microfluidic generation of semipermeable biomimetic polymer vesicles within 1 min using oleyl alcohol as the oil phase. The polymersomes allowed for the diffusion of protons and substrates acting as external signals. Using this method, we were able to construct adaptive microreactors containing internal polyelectrolyte-based catalytic organelles capable of sequestering and localizing enzymes and reaction products in a dynamic process driven by an external stimulus. This approach provides a platform for the rapid and efficient construction of robust adaptive microreactors that can be used in catalysis, biosensing, and cell mimicry.

## Introduction

Synthetic cell-like systems, such as protocells, artificial organelles, and microreactors, are a class of materials that exploit the compartmentalization principles of natural cells.^1−3^ The typical design of artificial cells is realized via a bottom-up approach to produce multicompartment systems with a compartment-in-compartment architecture that resembles the cytosolic organization of eukaryotic cells.^4−8^ In particular, the encapsulation of chemo- and biocatalysts in subcompartments enables the creation of cell-like microreactors for applications in biomimetic synthesis, molecular sensing, and biomedicine.^9−12^

Natural cells are versatile biological microreactors due to their ability to dynamically control biochemical processes through the assembly and localization of subcompartments and biocatalysts. They achieve this using biomolecular condensates of proteins and nucleic acids as highly dynamic compartments. The function, composition, and structure of these condensates are influenced by changes in cellular state and internal biochemistry.^13,14^ However, this high level of chemical control has not yet been reproduced in cell-like microreactors. Finding a combination of synthetic components that can reproduce dynamic compartmentalization without the biological machinery of cells is not trivial. In addition, assembling the various components into cohesive microreactors compounds the difficulties of creating functional, adaptive cell-like microreactors.

Biomimetic dynamic compartmentalization has been demonstrated in simple coacervate droplets formed by liquid–liquid phase separation (LLPS) of macromolecules, peptides, and nucleotides.^15−18^ Coacervates are characterized by a molecularly crowded microenvironment that allows selective sequestration of molecules from solution.^19,20^ The LLPS is driven by multiple weak noncovalent interactions that are easily perturbed, allowing these compartments to dynamically adapt to physical and chemical changes.^16,19,21^ The integration of coacervate droplets into lipid vesicles has been used as a method to study the dynamics of LLPS and biomolecule colocalization in multicompartmental systems.^22−26^ However, the development of methods and components that enable the fabrication of robust and adaptive microreactors for catalysts is still needed.

Although lipid vesicles are excellent biomimetic compartments due to their similarity to cellular vesicles, the methods required to create lipid-based multicompartment architectures can be challenging and time-consuming. For some applications, the lengthy process of up to several hours required by current microfluidic approaches can be undesirable when dealing with short-lived active biomolecules. Lipids are also susceptible to oxidative damage and colloidal instability, which can limit their applicability as compartments for microreactors.

Amphiphilic block copolymers offer an alternative to lipids. They can form compartments with customizable membrane chemistry and the desirable high mechanical stability needed to create robust synthetic cell-like systems.^15,27,28^ The high molecular weight of block copolymers results in vesicles with typically low permeability to hydrophilic molecules and ions.^29,30^ Although the integration of biopores and polymer design can mitigate such problems, the engineering of polymer-based biomimetic compartments with precise control over size, composition, and permeability remains an open challenge.^31−34^

In this work, we present a new design for an adaptive microreactor that combines polymer-based compartmentalization with dynamic coacervate-based colocalization and catalytic activity. We developed a novel microfluidic-based method that enables the assembly of ready-to-use adaptive multicompartment microreactors with a coacervate-in-vesicle structure within minutes. Unlike other microfluidic methods, the oleyl alcohol-assisted polymersome assembly (OAPA) method described in this work requires a minimal number of components: oleyl alcohol, a buffered medium, and an amphiphilic block copolymer. The specific combination of the organic phase and polymer leads to an immediate minimization of interfacial energies in the system, resulting in the formation of polymersomes within seconds. This robust method produced polymer vesicles with high throughput, controllable diameters, narrow size distribution, versatile encapsulation, and selective membrane permeability for small molecules, making it a robust and versatile platform for microreactor design.

OAPA allowed encapsulation of coacervate-forming components that undergo pH-responsive LLPS without a loss of vesicle integrity. The dynamic formation of internal coacervate droplets acted as adaptive artificial organelles that controlled the spatial organization and colocalization of enzymes, while allowing an external substrate to be processed internally through an organelle-localized cascade reaction. Overall, this study presents a method for constructing fully synthetic and adaptive synthetic microreactors capable of manipulating enzymatic reactions.

## Results and Discussion

### Formation of pGUVs by OAPA

**Figure 1:**
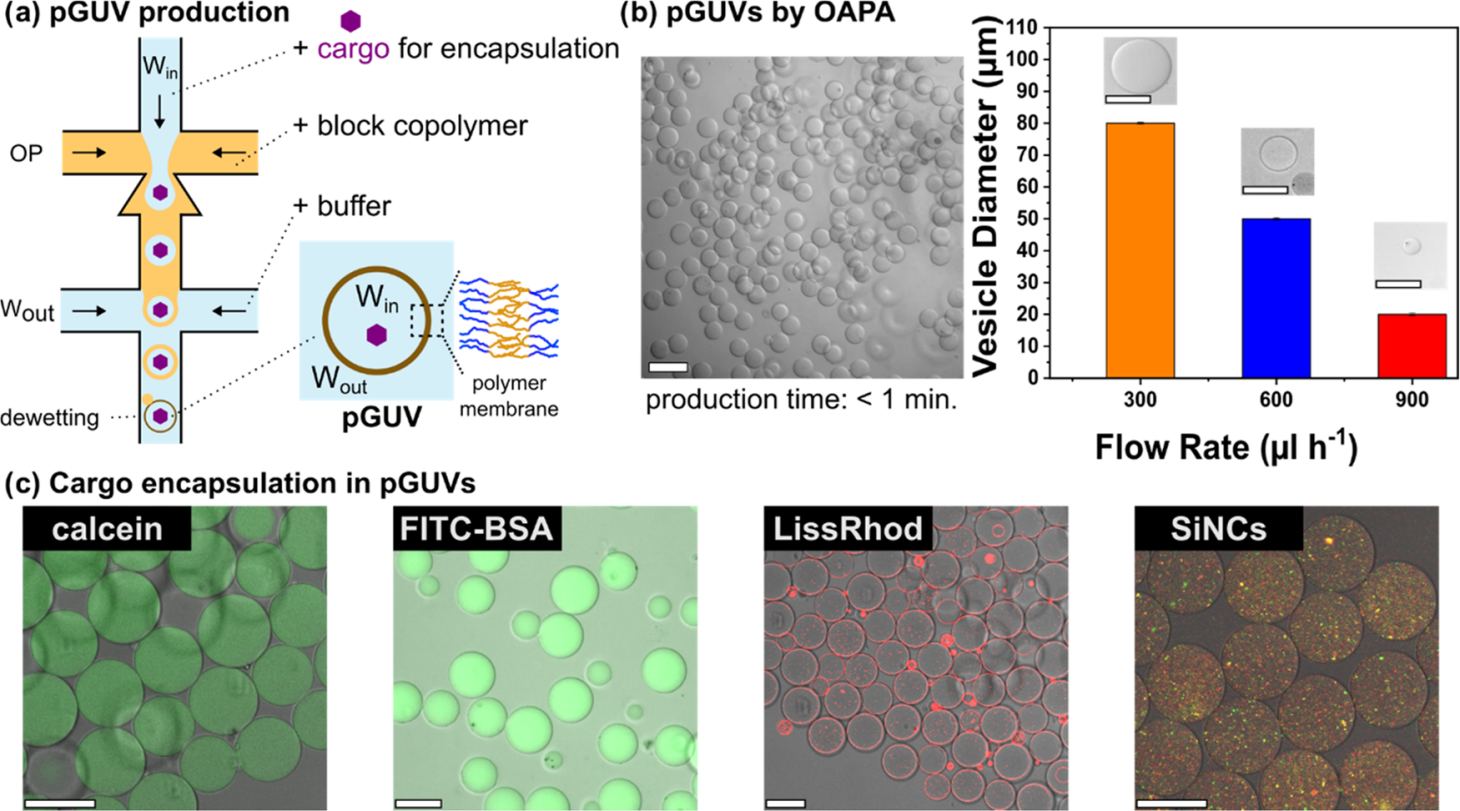
Microfluidic method for polymersome formation. (a) Schematic of polymersome formation in a microfluidic chip; (b) control of vesicle size by flow rate of outer aqueous phase. The vesicle production rate (kHz) is also shown. Standard deviations (SD) = 0.3 (1 kHz), 0.7 (2 kHz), and 1.9 μm (4 kHz). Scale bar: 50 μm. (c) Confocal microscopy images showing encapsulation of calcein (50 μg mL^–1^), FITC-BSA (0.1 mg mL^–1^), and labeled silica nanocapsules (10 wt %). Also shown is the incorporation of a lipid, LissRhodPE, into the vesicle membrane. Scale bars: 50 μm.

Polymeric giant unilamellar vesicles (pGUVs) were prepared by using droplet-based microfluidics. The microfluidic system included a PDMS chip with two junctions to generate double emulsion droplets (Figures S1 and S2). The composition of the fluids flowing through the chip consisted of an organic phase (OP) containing a dissolved membrane-forming low-molecular-weight amphiphilic diblock copolymer, poly(butadiene)_22_-poly(ethylene oxide)_14_ (*M*_n_ = 1200-*b*-600 g mol^–1^), an inner aqueous phase (W_in_) carrying the medium to be encapsulated, and an outer aqueous phase (W_out_) (Figure 1a). Polymeric unilamellar giant vesicles (pGUVs) were generated by forming a single emulsion at the first junction from aqueous droplets dispersed in the organic phase. These emulsion droplets were then passed through a second junction, where they were further dispersed by the external aqueous phase to form water-in-oil-in-water (W_in_/OP/W_out_) emulsion droplets. The final step resulted in vesicles and involved a dewetting transition, where excess oil was removed from the polymer membrane.

Our first goal was to develop a minimal formulation that did not require the use of additional surfactants during pGUV production. Common additives such as pluronic surfactants and poly(vinyl) alcohol can be incorporated into the membrane and cause undesirable effects such as reduced vesicle stability or nonspecific interactions with encapsulated materials.^35^ As shown previously, low-molecular-weight block copolymers can be used as macromolecular emulsifiers in the formation of double emulsions.^36^ Our hypothesis was that low-molecular-weight PB–PEO could also act as a sole emulsifier in the preparation of pGUVs by microfluidics.

First, we tested the effectiveness of several organic solvents that were compatible with the PDMS chips used for microfluidics. These were oleic acid, an oleic acid/isopropanol mixture, mineral oil, 1-octanol, and oleyl alcohol. PB–PEO was dissolved in the organic solvents at concentrations ranging from 1 to 20 mg mL^–1^. Images of vesicle formation are shown in Figure S3. The first organic phase tested was oleic acid, which is commonly used in liposome preparation. Oleic acid could be used to create thin-shell double emulsions in microfluidic chips. However, examination after 5–10 min showed only nondewetted double emulsions without fully formed polymersomes. Mineral oil produced complex double emulsions in an uncontrolled manner. The stability of the droplets was poor, and the vesicles burst quickly before dewetting could occur. The oleic acid/isopropyl alcohol mixture did not result in droplet formation at any of the junctions in the microfluidic chip.

The first pure-alcohol-based organic phase tested, 1-octanol, showed promising results. The formation of the double emulsions was stable. However, when the product was inspected after 5–10 min, mainly double emulsions or oil droplets were observed, with only a few pGUVs present. When the organic phase consisted of oleyl alcohol (Figure S4), a complete and rapid dewetting transition occurred in the resulting double emulsions, leading to the efficient formation of pGUVs (Figures S5 and S6). A large number of stable monodisperse vesicles (approximately 60 000) were obtained within 1 min of starting production. The vesicles were stable for at least 72 h after their formation (Figure S7). Interfacial tension (IFT) measurements of PB–PEO in oleyl alcohol showed that the IFT was below 2 mN m^–1^ at polymer concentrations above 5 mg mL^–1^ (Figure S8). Formulations with 10 mg mL^–1^ PB_22_–PEO_14_ in the OP produced an IFT of 0.2 mN m^–1^. This low IFT value was consistent with efficient dewetting observed during vesicle preparation. Two processes must take place during dewetting: adhesion of the two membrane leaflets and expulsion of the excess oil. For this to occur, the adhesion energy must be sufficient to overcome the energy barrier created by the interfacial tension (IFT) of the oil phase in water. Previous calculations for the analogous polymer PS–PEO have shown that the adhesion energy is approximately 1 mN m^–1^.^37^ Therefore, the low IFT values obtained with PB–PEO are consistent with the efficient dewetting observed during pGUV formation by OAPA. Our results are consistent with observations that alcohols can be effective cosurfactants.^38^ The disparity in molecular size between 1-octanol (C8) and PB–PEO (C44) is energetically disadvantageous and is expected to induce membrane defects. This leads to the formation of fragile vesicles that are unable to withstand the dewetting process, as confirmed by the low vesicle yield observed. Conversely, extended oleyl alcohol (C18) is more compatible with PB–PEO membranes. Indeed, a residual amount of oleyl alcohol persists within the vesicle membranes, supporting the theory that oleyl alcohol serves as a more suitable cosurfactant for the macromolecular surfactant PB–PEO.

The frequency and size distribution of vesicles obtained with the OAPA were measured at different flow rates (Figure 1b). The oil phase in all measurements consisted of 10 mg mL^–1^ PB_22_–PEO_14_ in oleyl alcohol. At the low flow rate of about 300 μL h^–1^, the frequency of double emulsion formation was about 1000 droplets per second (1 kHz). The droplets showed high size homogeneity (RSD = 0.34). At flow rates around 900 μL h^–1^, the frequency of droplet production increased to 4 kHz (RSD = 1.98) (Figure S9). The size of the resulting pGUVs could be controlled by adjusting the flow rate of the outer aqueous phase in the range of 20–80 μm.

Another aspect of the versatility of the vesicles produced by the OAPA products is their encapsulation capability and robustness. The ability to store hydrophilic cargo in the lumen of the vesicles is illustrated by the encapsulation of different types of materials (Figure 1c). As shown in the confocal microscopy images, all pGUVs loaded with calcein (20 μg mL^–1^) had similar internal compositions and size. The OAPA method also allows encapsulation of various biomolecules such as proteins, membrane-embedded hydrophobic molecules, and nanomaterials (Figure 1c). It was observed that the formation of pGUVs was disrupted when the concentration of encapsulated materials was too high. Specifically, the encapsulation of polyelectrolytes above a concentration of 20 mg mL^–1^ inhibited the formation of stable pGUVs.

### Equilibration of pH across the Membrane of pGUVs Obtained by OAPA

**Figure 2:**
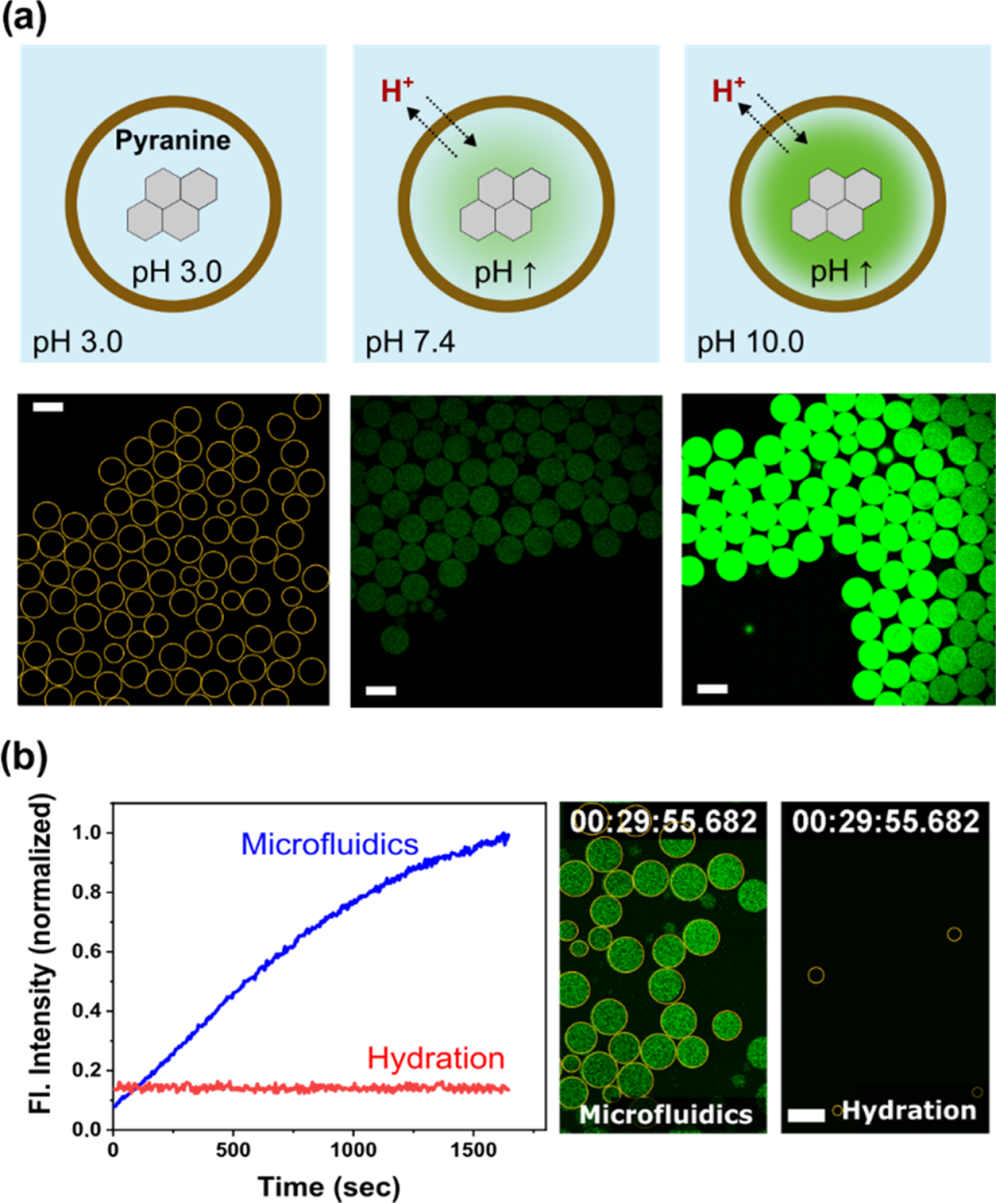
pH equilibration across the polymer membrane of pGUVs obtained by OAPA and film hydration; (a) Schematic and confocal images of vesicles containing 10 mM pyranine at acidic, physiological, and basic pH; (b) fluorescence intensity change of pyranine in pGUVs measured by confocal microscopy after external pH was increased to 8. Yellow contours were manually added to indicate the positions of the vesicles. Scale bar: 50 μm.

The pH equilibrium across the membrane of pGUVs was observed by confocal laser microscopy based on the changes in fluorescence intensity of the encapsulated pH-sensitive pyranine dye (Figure 2a). At basic pH (>8), the encapsulated pyranine has strong fluorescence emission, whereas at acidic pH (<7), it has low or no emission (Figure S10). To test the kinetics of pH equilibration across the vesicle membrane, pyranine was first encapsulated at pH 3 in pGUVs prepared by two different methods: OAPA and solvent-free hydration. The comparison of the results obtained by the two different methods allowed us to determine the effect caused by the possible presence of oleyl alcohol residues in the membrane of the vesicles obtained by OAPA. For comparison, oleyl alcohol-free pGUVs were obtained by thin layer hydration.^39^ A thin polymer film was cast on a solid surface by complete evaporation of the organic solvent (THF). The vesicles were formed after rehydration of the polymer film with buffer solution over a period of 24 h (Figure S11).

For both pGUV types, the external pH was adjusted to 8.0. The fluorescence intensity inside the pGUVs was measured over a period of 30 min immediately after the pH change. The OAPA vesicles showed a continuous increase in fluorescence intensity consistent with pH equilibration across the vesicle membrane (Figures 2b and S12). In contrast, vesicles obtained by film hydration showed no change in the internal pH under the same conditions. These results indicated that the difference in the permeability properties was likely a consequence of the presence of oleyl alcohol in the membrane of the pGUVs. HPLC measurements showed that between 0.9 and 1.4 wt % of the oleyl alcohol remained in the membrane of the vesicles obtained by OAPA (Table S1).

These results suggest that residual oleyl alcohol may have caused disruptions in the membrane layer that increased the permeability of the polymersomes. This hypothesis is consistent with the increased permeability observed in membranes perturbed by cross-linking of the polymeric membranes.^40^

### pH-Induced Coacervation in pGUVs

**Figure 3:**
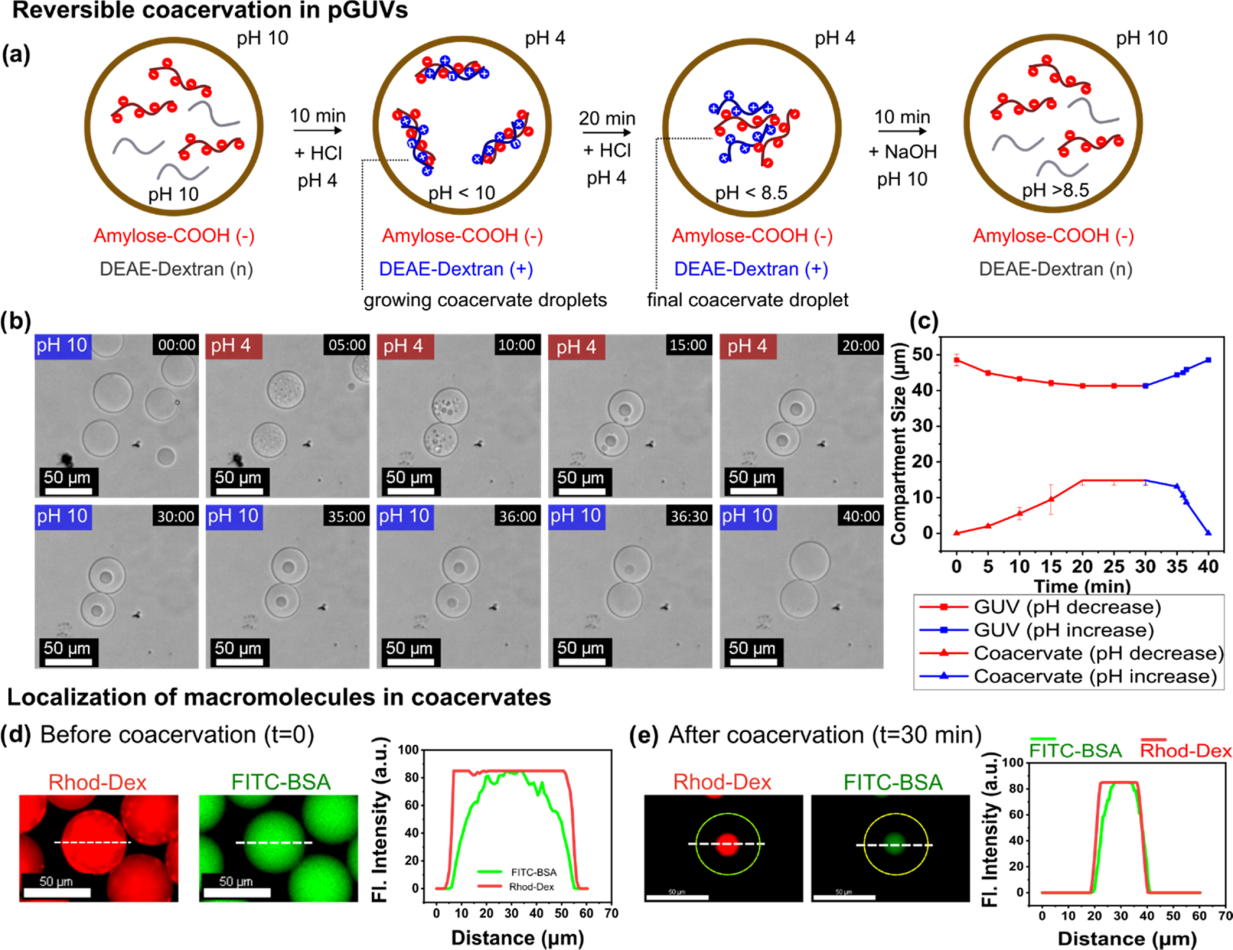
Coacervation in pH-controlled pGUVs; (a) Schematic representation of the coacervation process in pH-controlled pGUVs; (b) Confocal microscopy (CLSM) images showing coacervation over time. Internal composition: DEAE-dextran and amylose-COOH (5 mg mL^–1^ each) in 300 mM HEPES buffer and 50 μg mL^–1^ FITC-BSA. FITC-BSA was used for visualization only. External medium: 300 mM NaCl. (c) Change in compartment size at low pH; pGUVs initial size 48.5 μm (SD = 1.5) to final 41.3 μm (SD = 0.7) and coacervates from initial dispersed state to final 14.8 μm (SD = 1.3). (d) CLSM images and fluorescence profile showing the distribution of labeled macromolecules inside pGUVs at pH 10. (e) CLSM images showing the distribution of macromolecules in pGUV and internal coacervate droplet after 30 min at pH 4. The yellow contour indicates the border of pGUV, while the dotted line represents the plotted fluorescence profile. Scale bar = 50 μm.

The rapid pH equilibration across the membrane of pGUVs obtained by OAPA enables the use of these compartments for the construction of adaptive cell-like systems that respond to changes in pH (Figure 3a).^25,41,42^ This has been demonstrated by the formation of coacervate subcompartments within pGUVs. Coacervates are membraneless liquid-like droplets formed by liquid–liquid phase separation (LLPS) of components in solution. The droplets can sequester biomolecules, including proteins and nucleic acids, allowing the formation of artificial organelles.^23,43^

Coacervates were formed using two polyelectrolytes as components, carboxy-amylose (amylose-COOH) and diethylaminoethyl-dextran (DEAE-dextran) (Figures S13–S15). The components were encapsulated in pGUVs by the synthesis of aqueous OAPA. In bulk, coacervation produced distinct and stable liquid droplets when the pH was below 8.5 (Figures S16 and S17). The coacervates also formed in the presence of 100 mM sodium chloride (Figure S18), indicating high stability in the presence of electrolytes. Encapsulation of the coacervate-forming components in pGUVs was performed at pH 10 to maintain the uncharged state of DEAE-dextran and avoid premature coacervation (Figure S19). Both components were encapsulated at 5 mg mL^–1^ in a 300 mM HEPES buffer. After encapsulation, the pH of the outer medium was carefully lowered to 4. Coacervation was detected 5 min after the initial pH change (Figure 3b). Over a period of 20 min, the diameter of the pGUVs gradually decreased from approximately 48.5–41.3 μm, while the coacervate droplets grew to about 14.8 μm (Figures 3c and S20). The observed change in size of the pGUVs was attributed to the change in osmolality caused by coacervation. The coacervation within the pGUVs could be reversed by increasing the pH back to values above 10. The droplets were completely redissolved 10 min after the pH change. The process of coacervate formation and dissolution in pGUVs was successfully reproduced over a minimum of four cycles (Figure S21).

The pH-controlled coacervation inside pGUVs offers a versatile approach to control the localization of active components (e.g., enzymes, proteins, nucleic acids, substrates) inside the vesicles. To demonstrate this, the protein bovine serum albumin (BSA) was used as a model biomacromolecule. The protein was labeled with FITC dye for visualization and encapsulated in pGUVs together with rhodamine-labeled DEAE-dextran and unlabeled amylose-COOH (Figures S22 and S23).

At an internal pH above 10, both rhodamine-DEAE-dextran and FITC-BSA were homogeneously distributed in the internal volume of the polymersomes (Figure 3d). During the coacervation process, the protein was gradually entrapped in the resulting droplets. Upon completion of the coacervation process, the BSA was localized inside the droplet, as indicated by the overlapping fluorescence with labeled dextran (Figure 3e). Based on the fluorescence intensity measurements from CLSM, the partition coefficient of BSA within the coacervates was determined to be 176, consistent with efficient localization. The localization was reversed by raising the internal pH above 8.5. This approach allowed the adaptive formation of subcompartments, leading to a fine control of the internal structure, reminiscent of the behavior observed in cellular organelles.

### Membrane Permeability and Biomimetic Microreactors

**Figure 4:**
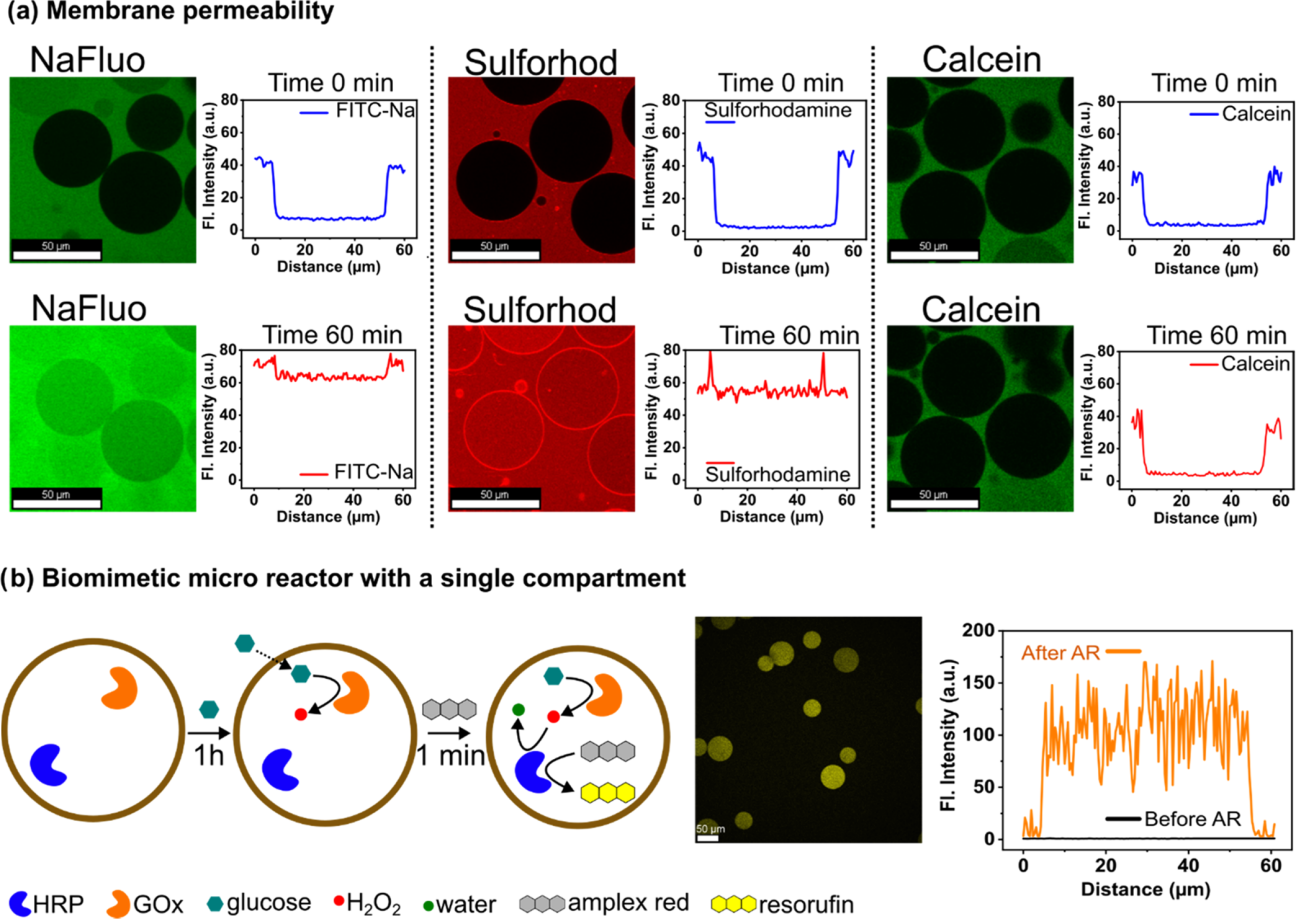
Permeability to molecules and biomimetic microreactor. (a) Membrane permeability was determined by CLSM over a period of 60 min. The profile plots show the fluorescence intensity along a line in the center of the vesicles. Scale bars: 50 μm. (b) Schematic representation of the structure and function of a biomimetic microreactor. The internal cascade reaction produces resorufin. The micrograph shows the fluorescence of resorufin produced inside a microreactor. The profile plot shows the intensity recorded before and after substrate addition. Scale bars: 50 μm.

We tested the membrane permeability of OAPA-derived pGUVs to various water-soluble molecules: fluorescein sodium salt (NaFluo), sulforhodamine B, calcein, and glucose (Figure 4a). The tests consisted of measuring the diffusion of the molecules through the polymer membrane by confocal laser microscopy over a period of 1 h. The dye with the lowest molecular weight, NaFluo (∼0.38 kDa), showed a relatively fast diffusion across the membrane. Sulforhodamine B (SRB, ∼0.55 kDa) can also diffuse in less than 1 h. Calcein (∼0.62 kDa), however, was unable to effectively cross the membrane in the same amount of time, indicating a threshold of 0.6 kDa (Figures 4a and S28). Larger molecules, such as Dextran at 4 and 10 kDa, were also unable to cross the membrane (Figure S25). Polymersomes prepared by the film hydration method showed no permeability to the same dyes (Figures S26 and S27).

The ability of vesicles to sequester biomolecules enables the development of microreactors for applications in (bio)chemical synthesis. To demonstrate this application, we constructed a microreactor consisting of two enzymes: glucose oxidase (GOx) and horseradish peroxidase (HRP). In this design, the enzymes were responsible for the chemical functionality of the microreactor, while pGUV provided structural support and intrinsic membrane permeability. Microreactors were created by encapsulating 50 μg mL^–1^ GOx and 8 μg mL^–1^ HRP in pGUVs using the OAPA method. The substrates for the enzymatic reaction, glucose and amplex red, were added externally (Figure 4b). First, glucose (100 mM) was added to the external medium, followed by a 1 h waiting period to allow the glucose (0.18 kDa) to cross the polymer membrane. Due to the intrinsic permeability of the pGUVs, glucose uptake across the semipermeable membrane occurs in less than 1 h (Figure S30). After glucose crosses the membrane, the reaction cascade is initiated by glucose oxidase to produce the intermediates hydrogen peroxide and d-(+)-gluconic acid-δ-lactone.

The second substrate (Amplex Red) was then added to the outer medium at a concentration of 25 μm mL^–1^. Its oxidation was catalyzed by horseradish peroxidase, resulting in the formation of resorufin (Figures 4b and S29). The entire process demonstrates the ability of pGUVs to retain active biomolecules and provide access to substrates.

**Figure 5:**
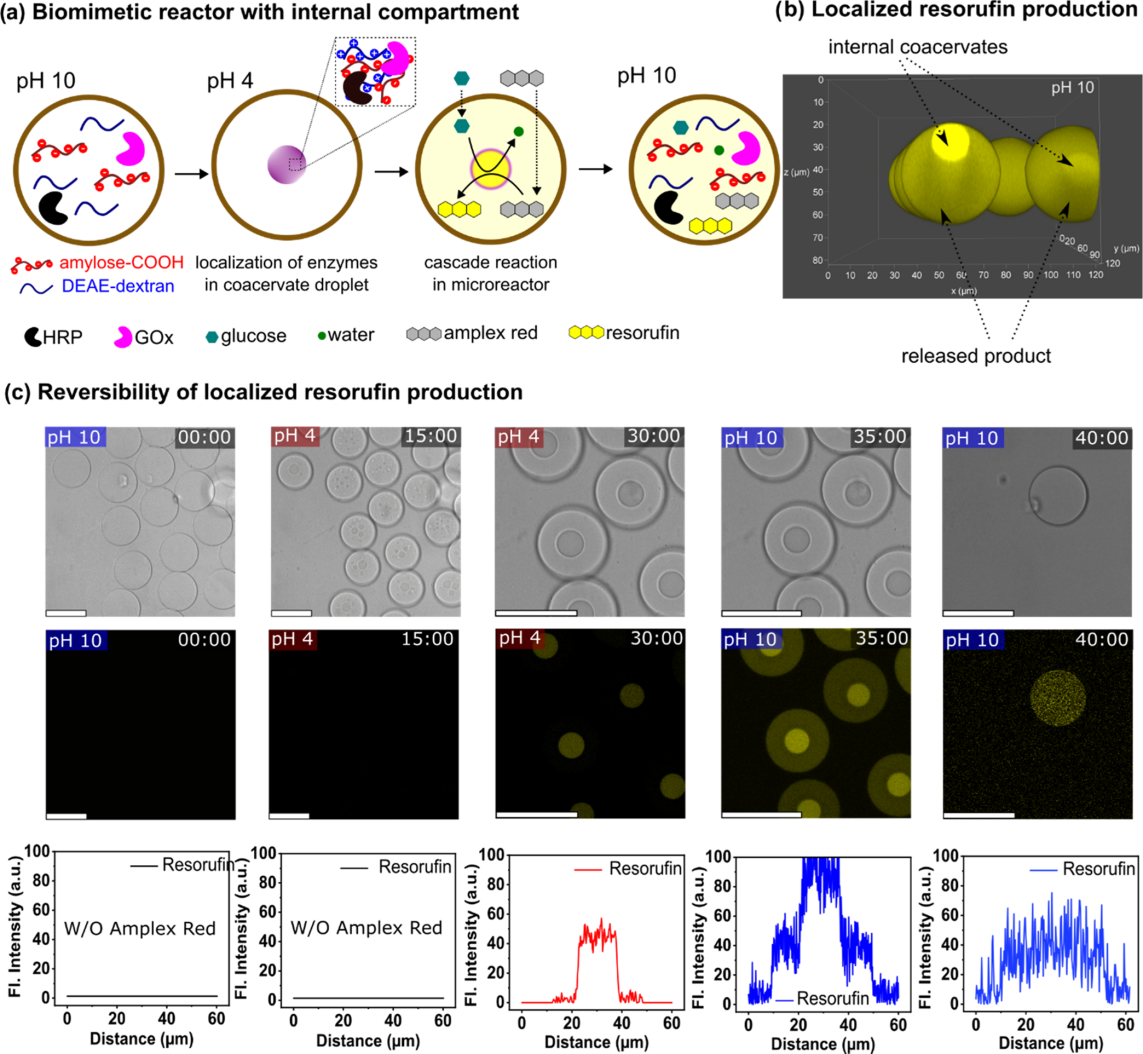
Biomimetic microreactor with dynamic compartments. (a) Schematic of microreactor design and effect of coacervation on enzyme and reaction product localization. (b) CLSM 3D reconstruction of fluorescence signal during localized production of resorufin in coacervate droplets encapsulated in pGUVs. (c) CLSM images showing the adaptive behavior of the microreactors during resorufin production over a 40 min period. Fluorescence profile plots show the distribution of resorufin in the vesicles. Scale bars = 50 μm.

The intrinsic permeability of the oleyl alcohol-assisted pGUVs allows chemical processes to be controlled by external stimuli. In principle, internal processes can go beyond simple cascade reactions if the active components can be efficiently encapsulated. We demonstrate this principle by incorporating dynamic compartmentalization into the enzymatic microreactors. In the new microreactor design, enzymes (50 μg mL^–1^ of GOx, 8 μg mL^–1^ of HRP) and the coacervation pair (amylose-COOH, DEAE-dextran, each 5 mg mL^–1^) were encapsulated in pGUVs at pH 10 (Figure 5a). At this pH, no coacervates could form, and the internal structure of the microreactor was similar to the monocompartment vesicle-based design (Figure 4b), with enzymes homogeneously distributed within the vesicles.

Lowering the pH to 4 resulted in the formation of coacervate subcompartments within the pGUVs. The coacervation process was gradual (Figure 5c). Initially, small droplets were formed that coalesced into a single coacervate droplet after approximately 30 min. Consistent with previous results (Figure 3), the coacervates inside the pGUVs were also able to sequester proteins in solution, resulting in their concentration being due to localization within the confined space defined by the droplet. The enzymes, GOx and HRP, were localized within the coacervate droplets, exhibiting partition coefficients of 48 and 83, respectively (Figure S24). The encapsulation and colocalization of HRP and GOx was also confirmed by the localized formation of resorufin observed when glucose and Amplex Red were added to the system. Glucose was added to the outer medium 30 min before the start of the coacervation process to allow it to completely penetrate the polymer membrane. Then, 25 μM Amplex Red in DMSO was added to the outer medium. The fluorescent reaction product (resorufin) was detected by confocal laser microscopy (Figures 5c and S30–S32). The results show that resorufin was formed mainly within the coacervate droplet. The presence of residual resorufin outside the droplets was consistent with the free diffusion of molecules due to the absence of a membrane around the coacervates. By readjusting the pH to 10, the coacervate began to gradually disintegrate, leading to the full release of the resorufin product into the internal medium of vesicle. The increase in the level of resorufin release is a direct result of pH regulation. This process is illustrated in Figure 5c. By externally changing the pH, we could control the localization of the components and the distribution of the product within the microreactor could be controlled.

## Conclusions

In summary, we developed a polymer-based microfluidic approach for the fabrication of dynamic multicompartment microreactors. This method allows the formation of polymer vesicles in large quantities with excellent control of vesicle size and versatile encapsulation. By exploiting the intrinsic permeability of the OAPA-generated polymer vesicles, we were able to create active multicompartment microreactors based on the confined liquid–liquid phase separation of polyelectrolytes triggered by external pH stimuli. In addition, we have shown that we can exploit the molecular sequestration properties of coacervate droplets for the dynamic localization of enzymatic reactions in polymeric microreactors. The microfluidics-based method provides a robust cell-like synthetic model for exploring the role of dynamic membrane-free subcompartmentation. Furthermore, the compatibility of the polymeric membrane and polyelectrolytes can serve as a basis for developing complex and dynamic synthetic cells with multiple compartments by incorporating additional stimuli-responsive modules. Overall, the method described in this work provides a versatile platform for the development of polymer-based cell-like systems, such as bioreactors and synthetic cells.

## Experimental Section

### Microfluidic Setup

The microfluidic chip design consists of an inlet channel for the three phases W/O/W and an outlet channel for the double emulsions (Figures S1 and S2). The double emulsions were created using a PDMS chip with two junctions. At the first junction, the inner aqueous phase is passed through the oil phase of the middle liquid, forming water-in-oil emulsions. These W/O emulsion droplets then flow through a central channel leading to the second junction, where the outer liquid phase is connected from either side of the channel. At the second junction or immediately thereafter, a double emulsion (water-in-oil-in-water) is formed and passed through the serpentine outlet channel via the outer liquid stream. The molds for the chips were fabricated using soft lithography techniques. The photolithographic process included the following steps: wafer cleaning, barrier layer formation, photoresist application, soft bake, mask alignment, exposure and development, and hard bake. To fabricate the microfluidic chips, the silicon wafers with the channel shapes were placed in a glass dish and covered with a 5–10 mm high mixture of liquid PDMS and curing agent in a ratio of 9:1 (approximately 40 mg of the mixture was required for one glass dish). The glass dish containing the wafer, PDMS, and curing agent was degassed using a conventional vacuum pump and chamber until all air bubbles were removed. The wafers were then stored at 80 °C for at least 2 h to promote molecular cross-linking. After curing, individual PDMS replicas were cut out with a scalpel. The channels were pierced with a syringe needle to create the inlet and outlet connections. A large coverslip was thoroughly cleaned with 70% ethanol. The glass slide, together with the PDMS replica (channels from the top of the device), was then activated in a plasma cleaner for 1 min at 20% radiofrequency (RF). The activated glass slide was then gently pressed onto the activated side of the PDMS replica with the microfluidic channels, and the channels were coated with a 5% w/w PVA solution and finally baked at 80 °C for at least 1 h.

### Microfluidics Formation of Polymersomes

The solutions prepared according to the previous section were aspirated into 1 mL plastic syringes and connected to the inlets of the microfluidic chip via a needle and tubing. The syringes and tubing were then checked for air bubbles, and if any were present, the chip was disconnected, and the bubbles were manually expelled. The chip outlet was then connected by tubing to an Eppendorf tube or directly to a dewetting chip. Syringes were attached to the microfluidic pump system, and the chip was placed over a microscope objective. Stable vesicles were generated by gradually adjusting the flow rates of the outer, middle, and inner fluids. Production was initiated by applying flow rates of 100 μL h^–1^ for the inner and middle fluids. Once the fluids reached the chip, the outer fluid was initiated at a rate of 200 μL h^–1^. Once the microfluidic chip was successfully initiated, the flow rates were adjusted to the desired values. The vesicles were discarded until the behavior of the double emulsion was stabilized at the second junction. Once equilibrium was reached, the double emulsion chip was connected to one or more dewetting chips. There the emulsions were completely dewetted and collected via a tube into a vial for further analysis.

### Solvent-Free Hydration Method for the Preparation of pGUVs

The PB_22_–PEO_14_ membrane component was dissolved in tetrahydrofuran (THF) at a concentration between 0.1 and 2%, corresponding to the concentrations used in the microfluidic method. The polymer solution was then transferred to a glass microscope chamber and left for 1 h in a fume hood under a slight flow of nitrogen. After a dry polymer layer was formed, 400 μL of a buffer solution at the desired pH and solute concentration was added. The solution was allowed to sit for 24 h to allow the formation of GUVs by hydration. After 24 h, the vesicles were separated from the polymer layer by very gentle pipetting and the pGUVs were examined under the microscope to determine their size and unilamellarity.

### Double Emulsion Droplet Method for the Preparation of pGUVs

Giant polymersomes for the permeability assay were prepared by a modified double emulsion method. First, 10 mg mL^–1^ of polybutadiene-*b*-poly(ethylene oxide) (PB–PEO) was dissolved in toluene at 600 rpm for 1 h. The inner solution consisted of a HEPES buffer solution at a concentration of 300 mM before the formation of the giant polymersomes. Then, 5 μL of the inner solution was quickly added to the solution consisting of the membrane component in toluene, which was gently mixed for about 1 min for emulsification. Then, 5 μL of the emulsified solution was quickly transferred to 200 μL of the outer solution (300 mM HEPES) and mixed by pipetting for about 1 min. The resulting solution was kept in a fume hood for ∼2 h to allow the toluene solution to evaporate. The final solution consisted of polymeric GUVs only.^36^

### High-Performance Liquid Chromatography (HPLC) Measurements of Oleyl Alcohol Content in the Membrane

The measurement was performed by a chromatographic experiment. The sample containing the polymersomes was freeze-dried for 24 h to remove water from the composition. The contents of the sample were dissolved in THF. High-performance liquid chromatography-ultraviolet (HPLC-UV) was performed using 10 μL of the sample dissolved in THF at 20 °C. The setup consisted of a 7725i injection valve with a 20 μL Rheodyne loop, a PSS degasser, an Agilent Technologies 1100 series quaternary gradient pump, an Agilent Technologies 1200 series column oven, an Agilent Technologies 1200 series photodiode array detector (DAD), and a 385-LC Varian ELSD detector. Results were obtained and analyzed by using Agilent OpenLAB CDS Chemstation software.

### Spinning Drop IFT Measurements

The measurements were performed by using the SVT 20N Spinning Drop Tensiometer from DataPhysics. The measuring cylinder was filled with the higher-density liquid, and then 100 μL of the lower-density liquid was injected through the lid using a glass syringe. The cylinder was then placed on the spinning system, and the measurement was performed at 8000 rpm at 25 °C and the data was analyzed using the system software.

### Synthesis of Carboxy-Amylose

Carboxy-functionalized amylose was prepared by dissolving 1.5 g of amylose and 3.6 g of NaOH in 15 mL of Milli-Q at 70 °C. After complete dissolution of the amylose, 2.7 g of chloroacetic acid was added and the reaction mixture was stirred for 2 h. After the reaction, the mixture was neutralized with acetic acid and precipitated in 200 mL of cold ethanol. The resulting precipitate was dissolved in Milli-Q water and dialyzed extensively against water using regenerated cellulose dialysis tubing (Spectrum Laboratories) with an MWCO of 3.5 kDa before freeze-drying. 1H NMR (D2O) characterization data and chemical structures are shown in Figure S16.^20,33^

### Enzymatic Cascade Reactions in pGUVs

The PB–PEO polymersome system was assembled by using droplet-based microfluidics. The internal volume of the polymersomes consisted of 5 mg mL^–1^ of both DEAE-dextran (*M*_w_ = 10 000 g mol^–1^) and amylose-COOH (*M*_w_ = 15 000 g mol^–1^) coacervate pairs, the reaction enzymes GOx and HRP at concentrations of 50 μg mL^–1^ GOx and 8 μg mL^–1^ HRP in 300 mM HEPES buffer at pH 10. Immediately after the formation of the polymersomes in the microfluidic chip, a large number of polymersomes are introduced into a microscope chamber containing 300 mM NaCl solution. The sodium chloride solution was chosen to create a density difference between the inner volume of the polymersomes and the outer medium, thus causing the GUVs to sink to the bottom of the chamber. The pH of the HEPES is then adjusted to 5 to induce phase separation within the GUVs via the charged DEAE-dextran and amylose-COOH and the formation of coacervate droplets. The cascade of reactions is initiated by adding 100 mM glucose to the outer medium followed by a period of ∼1 h to allow the glucose to diffuse through the polymer membrane into the vesicles. The glucose is consumed by the GOx enzyme in the coacervate droplet, producing the intermediates hydrogen peroxide and d-(+)-gluconic acid-δ-lactone, which saturate the volume of the coacervate droplet. Immediately after the addition of 25 μL mL^–1^ Amplex Red dissolved in DMSO to the outer medium, the enzymatic reaction completes the production of fluorescent resorufin in the coacervate droplet in the GUVs. To release the resorufin throughout the internal volume of the polymersome, the pH of the outer medium was again adjusted to pH 10 and the coacervate droplet was dissolved and the resorufin was distributed throughout the GUV in less than 5 min. The entire reaction cascade was performed at an ambient temperature of 18–21 °C.

### Microscopic Analysis

Light microscopic analysis was performed using a Leica DMi 8 inverted microscope equipped with a Vision Research Phantom VEO 710 high-speed camera. The high-speed camera was controlled by dedicated software (Phantom Camera Control Application PCC 2.6, Vision Research). Confocal laser scanning microscopy (CLSM) images and videos were acquired using a Leica TCS SP5 II system with four solid-state lasers with excitation wavelengths of argon, DPPS 561 nm, HeNe 594, and HeNe 633 nm. The accompanying LAS X software enabled image acquisition and processing. Images were acquired using immersion objectives for 25.0 × 0.95 water and 63 × 1.20 water UV magnification.

## Supporting Information Available

Additional experimental details; materials; and characterization data including images of the experimental setup (PDF)
Production of double emulsions by OAPA (Movie S1) (AVI)
Dewetting of double emulsions by OAPA (Movie S2) (AVI)
Coacervation in pGUVs at low pH (Movie S3) (AVI)
Coacervate disassembly in pGUVs at high pH (Movie S4) (AVI)
Dynamic localization of macromolecules in pGUVs (Movie S5) (AVI)

## Supplementary Material

am3c09196_si_001.pdf

am3c09196_si_002.avi

am3c09196_si_003.avi

am3c09196_si_004.avi

am3c09196_si_005.avi

am3c09196_si_006.avi
