# Supplementary material for: Polymeric Microreactors with pH-Controlled Spatial Localization of Cascade Reactions
Source: ACS Appl Mater Interfaces. 2023 Oct 30;15(44):50755–64. doi: 10.1021/acsami.3c09196 (PMC10636718; doi:10.1021/acsami.3c09196)
Supplement: Supplementary file 1 — am3c09196_si_001.pdf [file am3c09196_si_001.pdf]

Supporting Information

Polymeric Microreactors with pH-controlled Spatial Localization of Cascade Reactions

Tsvetomir Ivanov, Shoupeng Cao, Nitin Bohra, Marina de Souza Melchiors, Lucas Caire da Silva, Katharina Landfester**

Department of Physical Chemistry of Polymers

Max Planck Institute for Polymer Research

Ackermannweg 10, 55128 Mainz (Germany)

E-mail: silva@mpip-mainz.mpg.de

landfester@mpip-mainz.mpg.de

Experimental Procedures

1.1 Materials

Sucrose, D-(+)-Glucose, Oleyl alcohol (techn. Grade 85%), Mineral oil, Propanol, 1-octanol, F108 surfactant, F68 surfactant (sold under the commercial name Poloxamer 188), poly(vinyl alcohol) (PVA) (Mw 9000-10000, 80 % hydrolized), Polyethylene glycol (PEG) (MW 6000), Bovine serum albumin (lyophilized powder >96 %) , Diethylaminoethyl-dextran -hydrochlorid, Horseradish peroxidase (lyophilized, powder, ~150 U.mg-1), Glucose oxidase (Type VII, lyophilized powder, ≥100,000 units/g solid), Sulforhodamine B (Dye content 75 %), Calcein, Fluorescein sodium salt, Amplex Red and HEPES solution 1M, pH 7.0-7.6 were all purchased from Sigma Aldrich. Sodium chloride and 3-morpholinopropane-1-sulfonic acid (MOPS) were both purchased from Carl Roth GmbH & Co. Kg., while oleic acid was purchased from Acros Organics. HCl (≥32 %), ethanol (≥ 99.9 %, ethanol for analysis), chloroform (≥ 99.8 %) were purchased from Merck KGaA. Furthermore, polydimethylsilxane (PDMS, SYLGARD silicone elastomer) and its curing agent (SYLGARD silicone elastomer curing agent) were obtained from DOW CORNING, red fluorescent 1,2-dioleoyl-sn-glycero-3-phosphoethanolamine-N- (lissamine rhodamine B sulfonyl) (Liss Rhod PE) were all obtained from Avanti Polar Lipids. PB-PEO

polymers (P41745-BdEO – Mn 1.2-b-0.6 kg mol-1) were purchased from Polymer Source Inc.

Ultrapure water was obtained using a Milli-Q R Advantage A10 water purification system.

Supplementary Figures

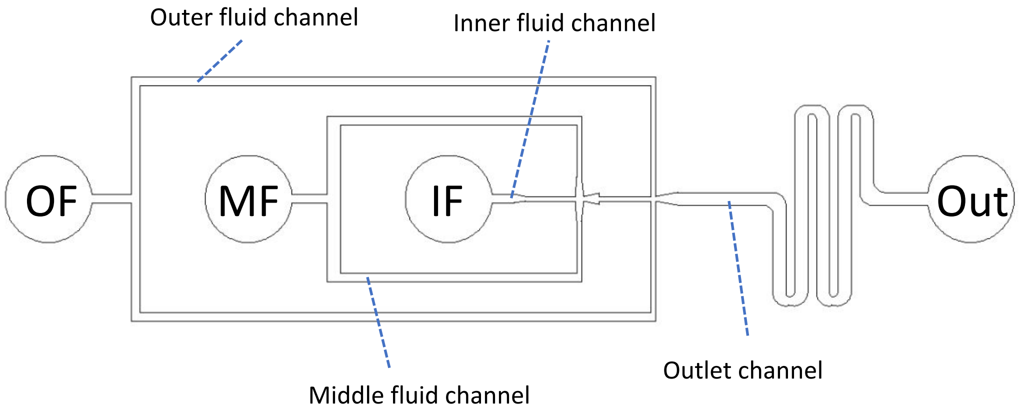

Figure S1. Construction of a chip for the formation of double emulsions. Consisting of 3 inlets for the two water phases and the organic phase, followed by a serpentine outer channel to promote dewetting.

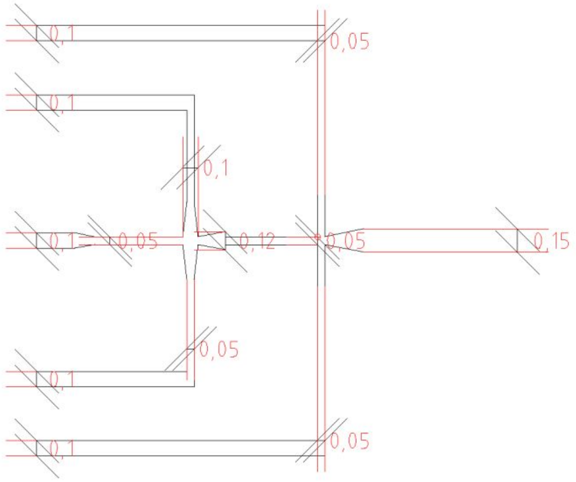

Figure S2. Dimensions of the double emulsion chip in micrometers. The width of the channels was 0.1 mm, while the two junctions were 0.05 mm wide. The outlet channels had the largest width with 0.15 mm. The height of the channels was about 0.5 mm.

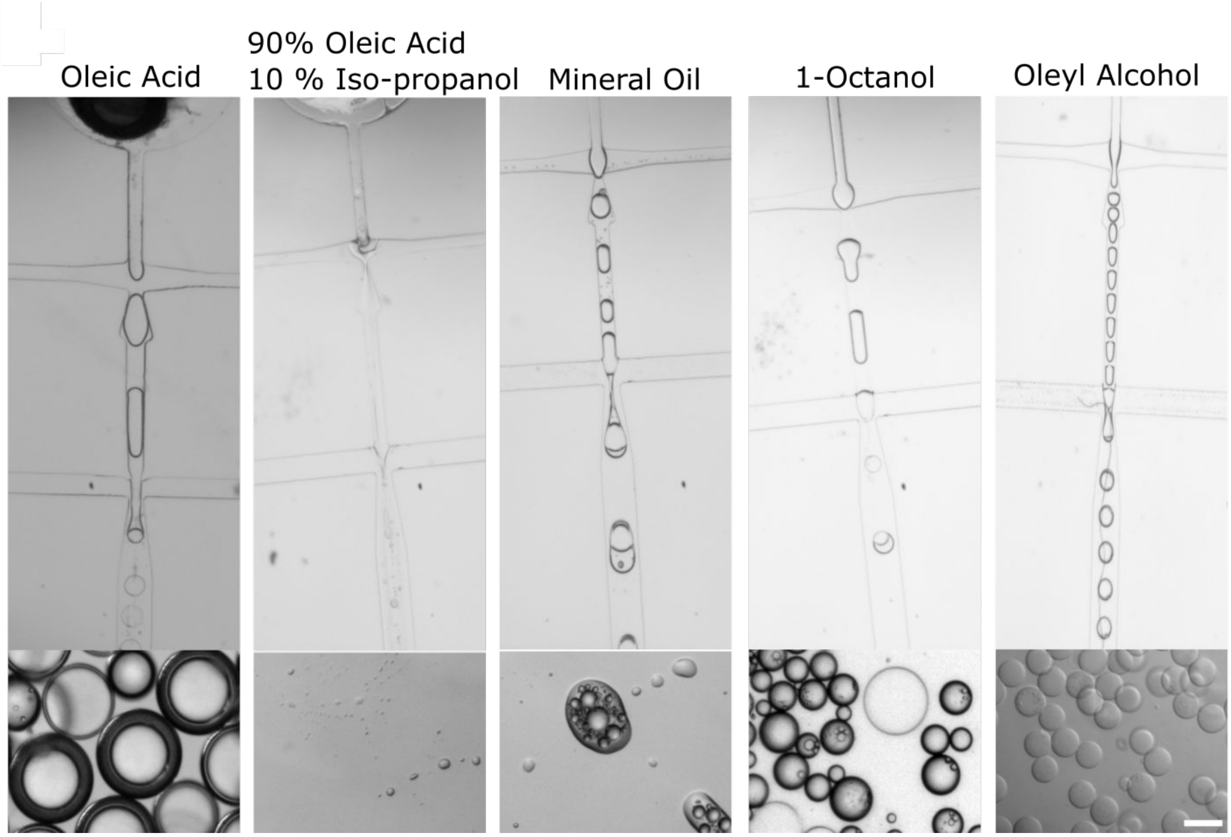

Figure S3. Production of double emulsions. Organic phase: 10 mg ml^{-1} PB_{22}-PEO_{14} dissolved in different organic solvents. The last row shows bright-field images of the materials prepared with each organic phase. Scale bar (applies to all images) = 50 μm.

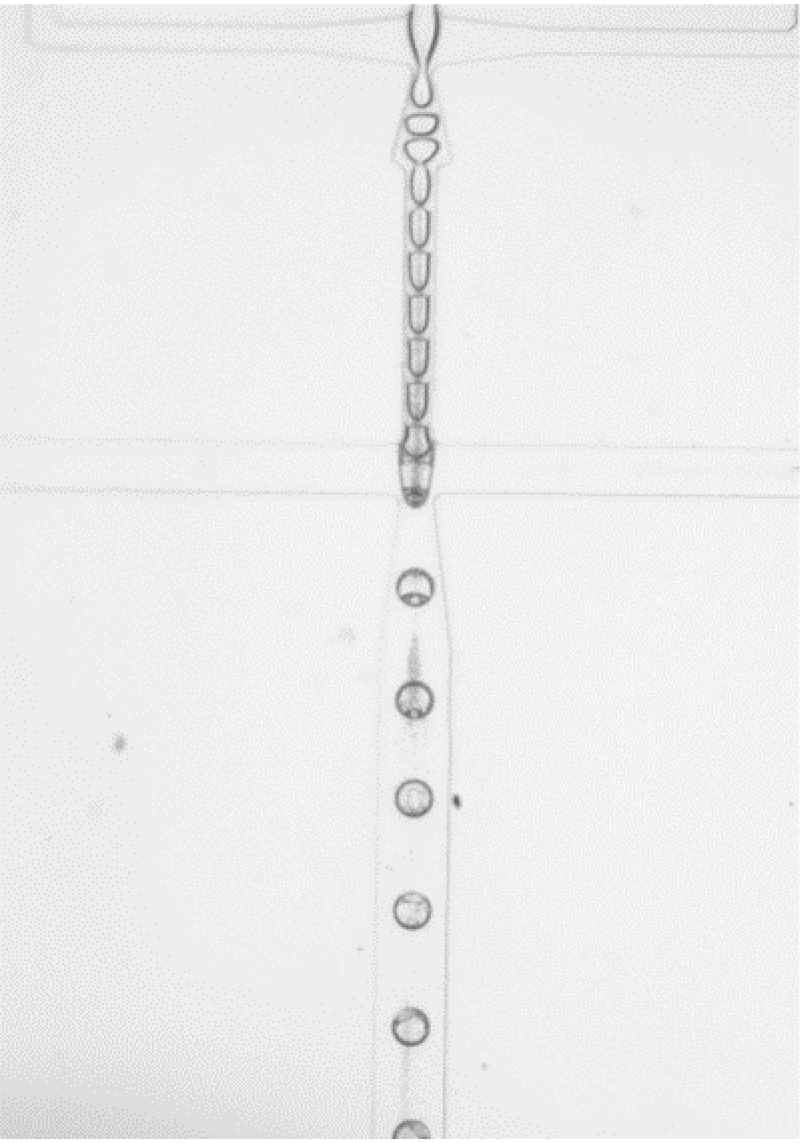

Figure S4. Production of double emulsions with oleyl alcohol. Organic fluid composition: 10 $mg.ml^{-1}$ PB_{22}-PEO_{14} in oleyl alcohol. Flow rates: inner fluid 40 $\mu l.h^{-1}$, middle fluid 40 $\mu l.h^{-1}$and outer fluid 400 $\mu l.h^{-1}$. Images taken with a high-speed camera.

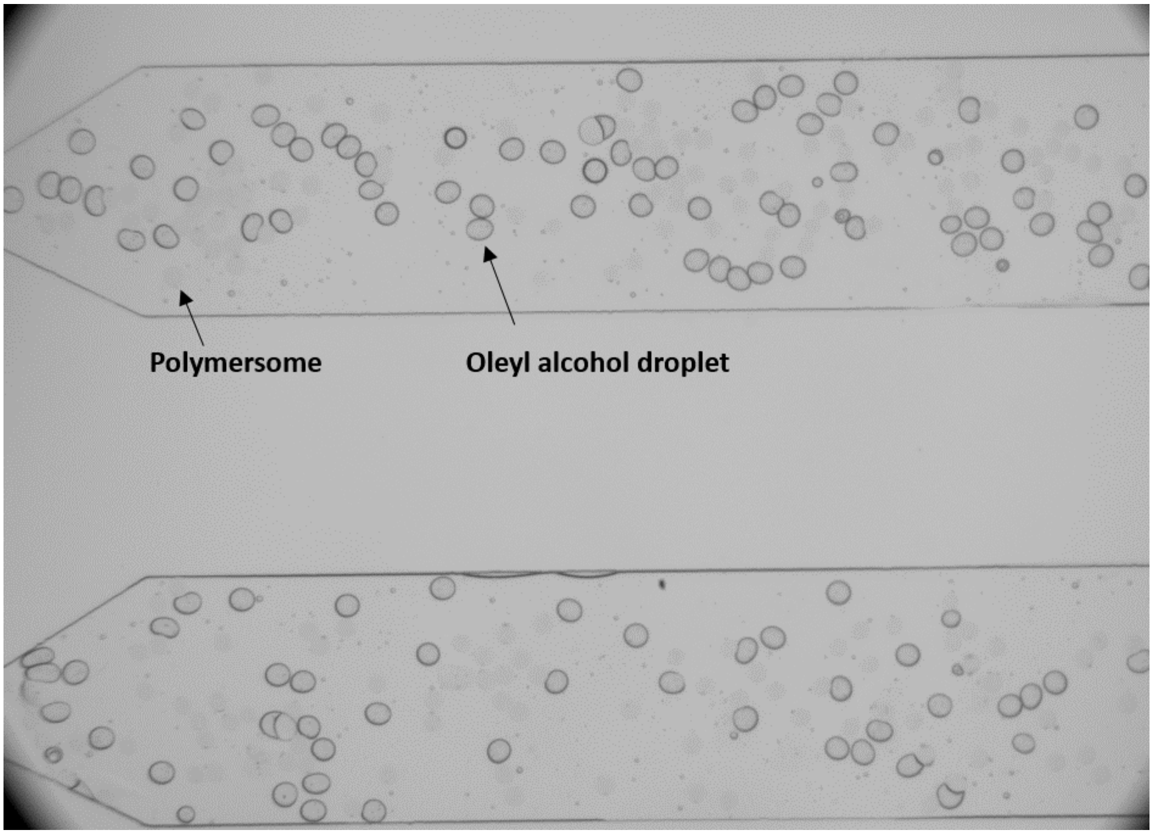

Figure S5. Dewetted vesicles immediately after preparation in a dewetting chip with spiral design 3.00 mm in length and 0.5 mm in width. The large chamber was specially designed and connected to the double emulsion chip, where the double emulsions are prepared and directly transferred for testing of dewetting. The high-contrast droplets are the organic phase oleyl alcohol, while the smaller translucent circles represent the polymersomes. High-speed camera imaging.

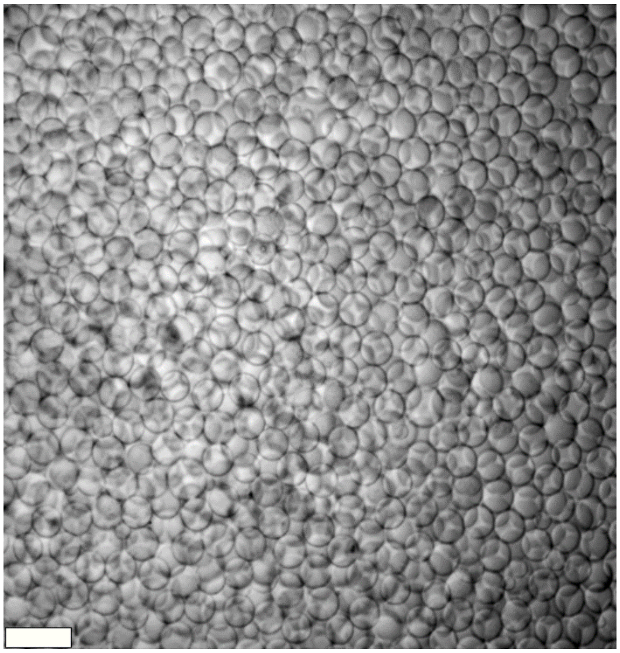

Figure S6. PB_{22}-PEO_{14} vesicles dispersed in 1 μl of outer fluid immediately after formation. Composition: 300 mM HEPES buffer in the inner volume and 300 mM NaCl in the outer medium. Bright-field imaging. Scale bar = 100 μm.

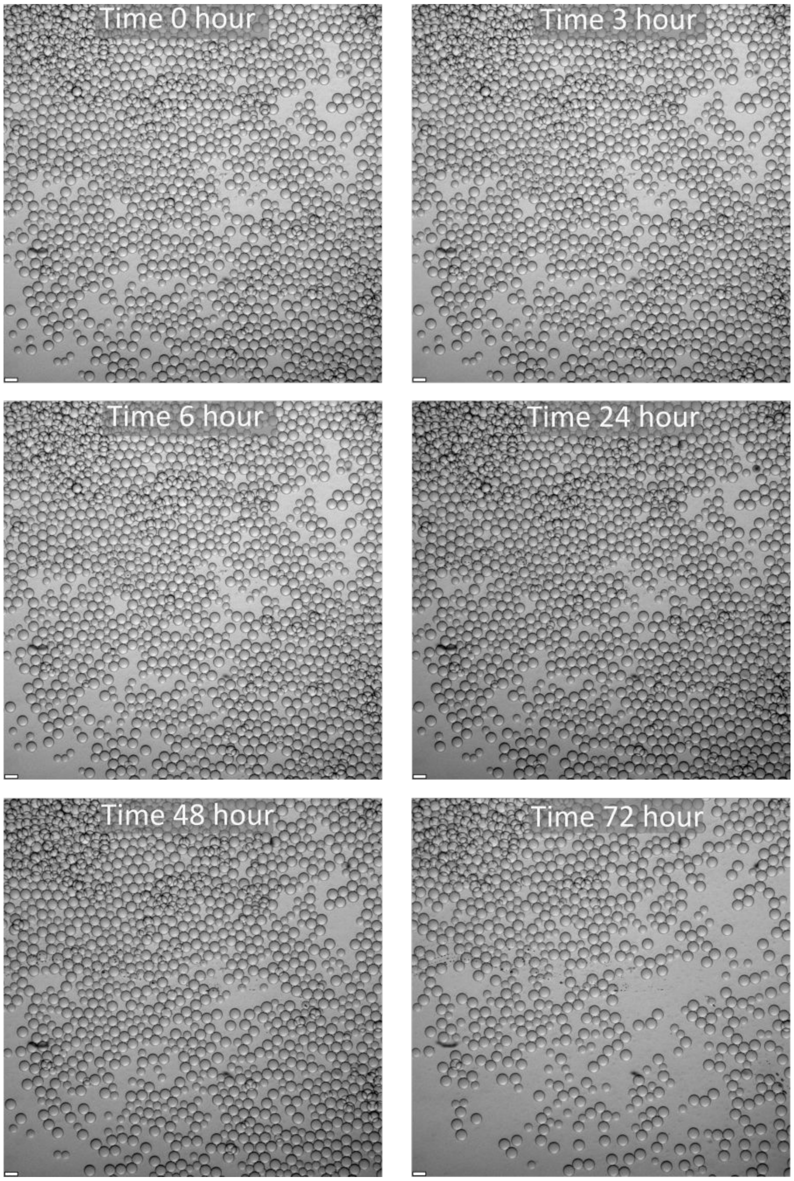

Figure S7. Stability study of PB_{22}-PEO_{14} vesicles dispersed in outer fluid over time. The analysis was done by capturing images of the polymersome-containing microscope chamber at every hour for a period of 6 hours and then additional images at 24, 48 and 72 hours. The stability was

calculated as percentage of the number of initial polymersomes at the start and the final number of polymersomes at 72 hours, which was approximately 86 %. Scale bar = 50 µm.

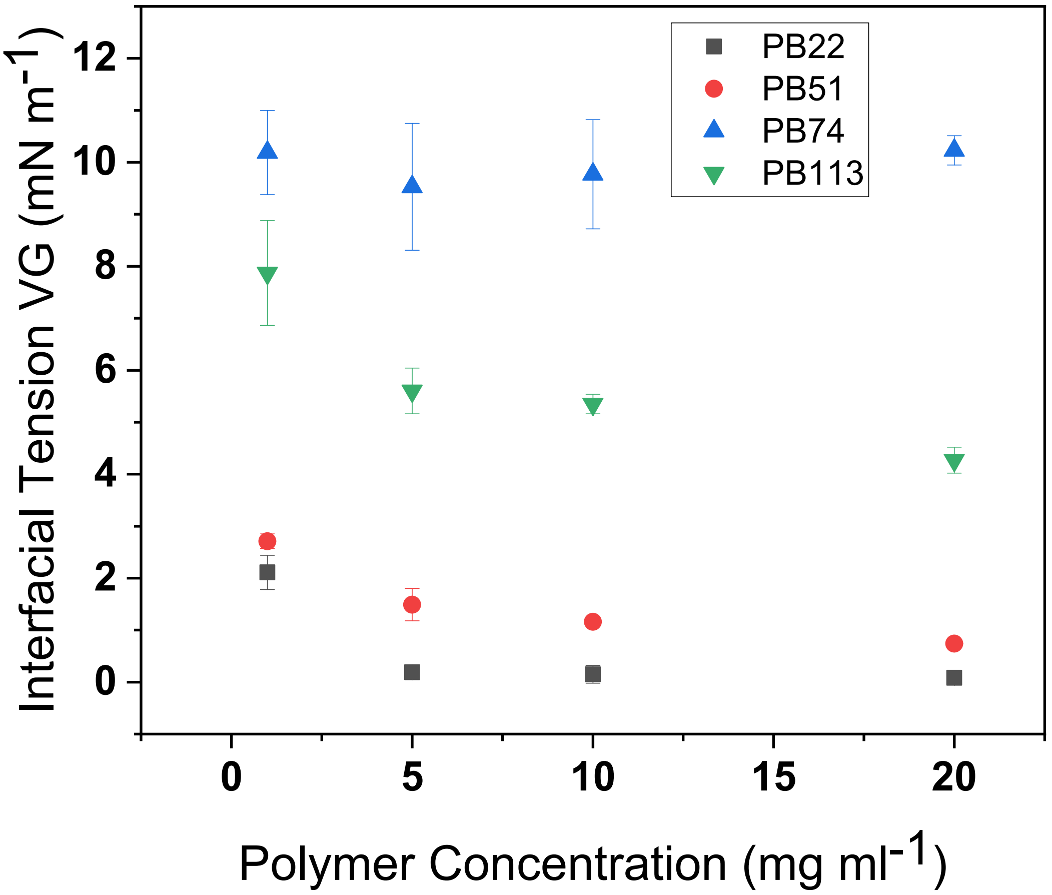

Figure S8. Interfacial tensions between water and oleyl alcohol at different concentrations and block lengths of PB-PEO. The following polymer compositions were used: PB_{22}-PEO_{14}, PB_{51}-PEO_{28}, PB_{74}-PEO_{35}, PB_{113}-PEO_{75}. The results were acquired through spinning drop tensiometry.

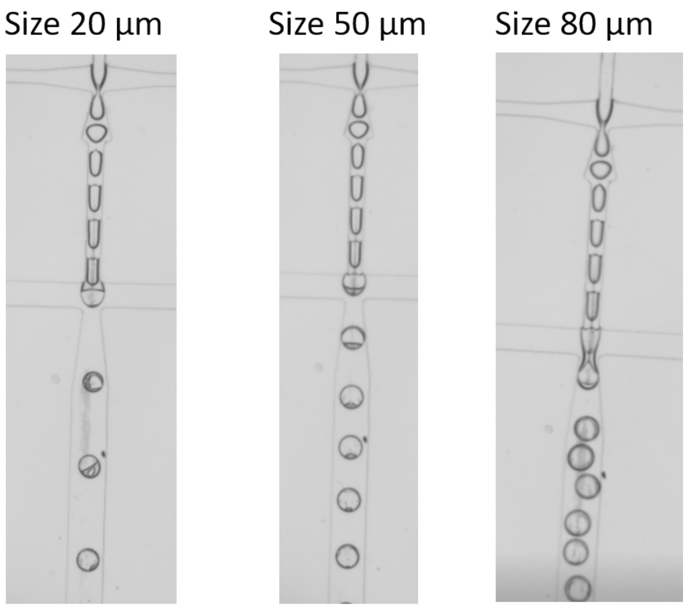

Figure S9. Formation of pGUVs with final diameters controlled by the flow rate of the outer fluid. Composition of the oil phase: PB_{22}-PEO_{14} (10 mg mL^{-1}) in oleyl alcohol. 20 µm (900 µL h^{-1}), 50 µm (600 µL h^{-1}) and 80 µm (300 µL h^{-1}). Images were taken with a high-speed camera.

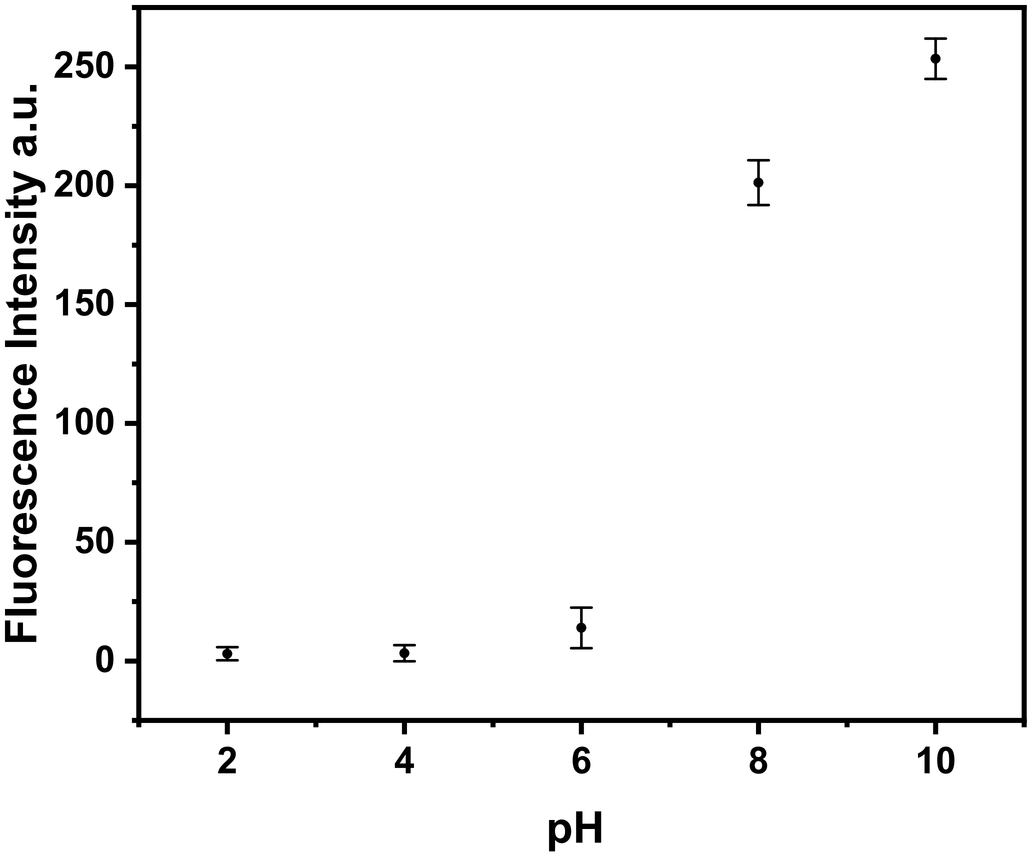

Figure S10. Fluorescence intensity of 10 mM pyranine at different pH values in 50 mM HEPES solution. The pH responsive dye showed almost no fluorescent emission at pH values below 7, while it emitted strongly at basic pH. Data obtained by confocal laser scanning microscopy.

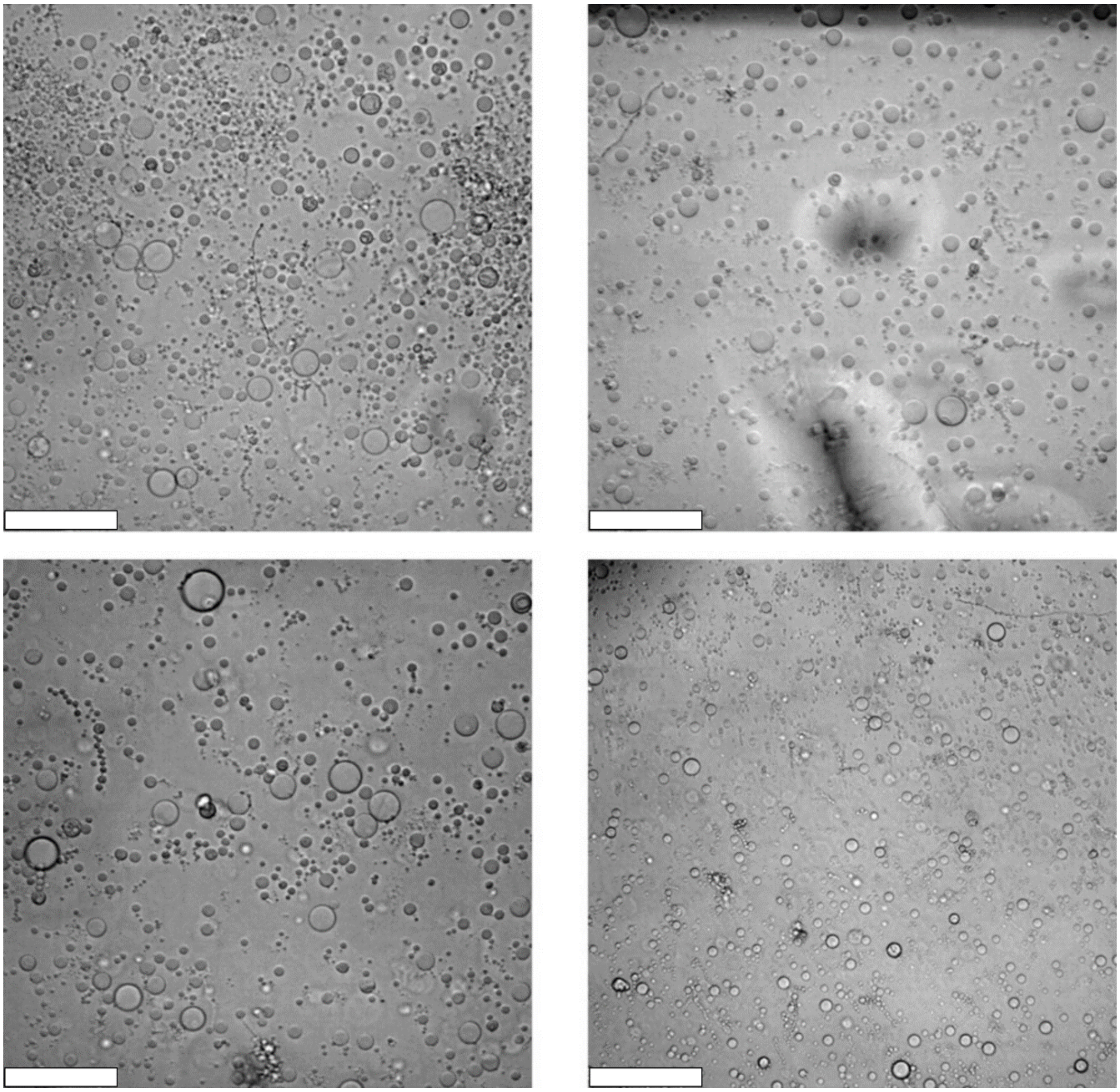

Figure S11. PB_{22}-PEO_{14} pGUVs produced by the film hydration method. The film was hydrated with a 30 mM HEPES buffer. Scale bar = 100 μm. Bright-field images.

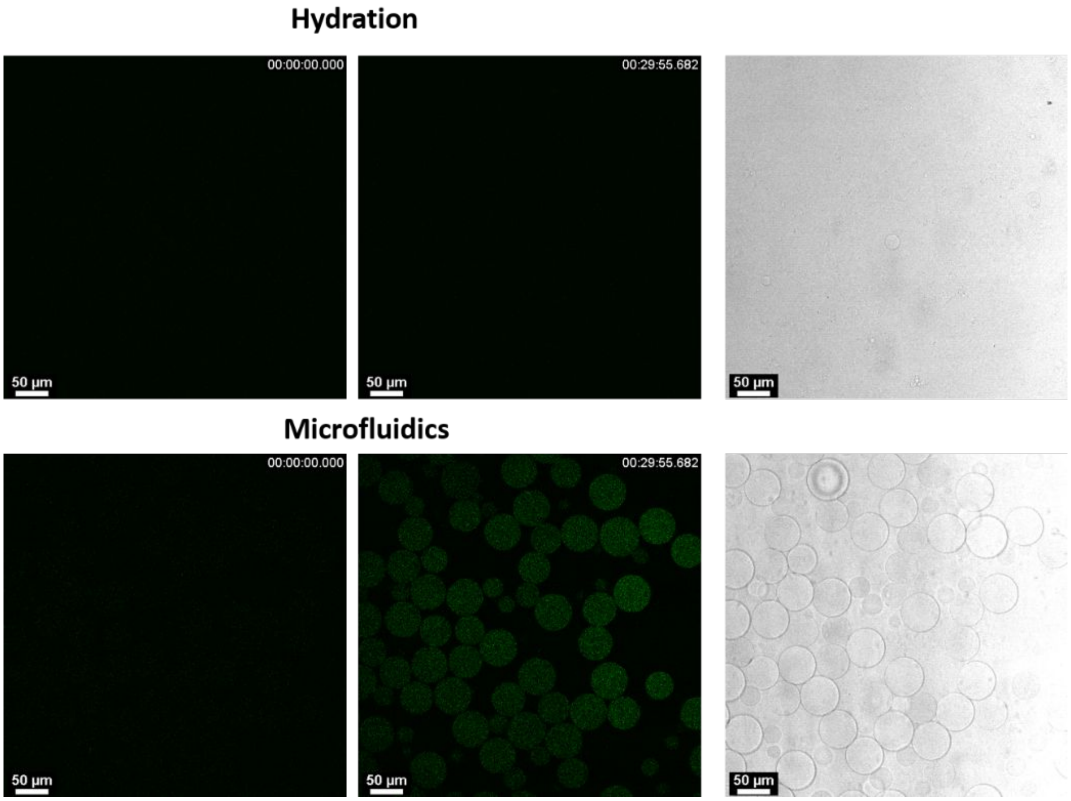

Figure S12. Encapsulation of the pH-responsive dye pyranine (10 mM) in pGUVs prepared by the hydration method and OAPA. Fluorescence intensity immediately after pH change and 30 minutes thereafter. The results show that pH equilibration did not occur with pGUVs obtained by hydration. In contrast, pGUVs obtained by OAPA showed a significant change in fluorescence intensity. Data obtained by confocal laser scanning microscopy.

Table S1. Data showing the experimental conditions and the results of HPLC measurements on the content of oleyl alcohol in the final membrane composition of PB22-PEO14 pGUVs. The solvent used for the measurements was THF. The final concentration of oleyl alcohol in the membrane ranges from 0.87-1.37%.

Sample	Conc. Sample g/l in THF	Inj, Volume µl]	UV Surface 200 nm	ELSD Surface	UV Conc. OA g/l	UV Solid %
OA1	4.04	10	426.028	9.337 e^{-5}	0.055235252	1.367
OA1	-	10	426.969	9.53 e^{-5}	-	-
OA2	2.71	10	171.26	N/A	0.023622094	0.87
OA2	-	10	193.535	N/A	-	-

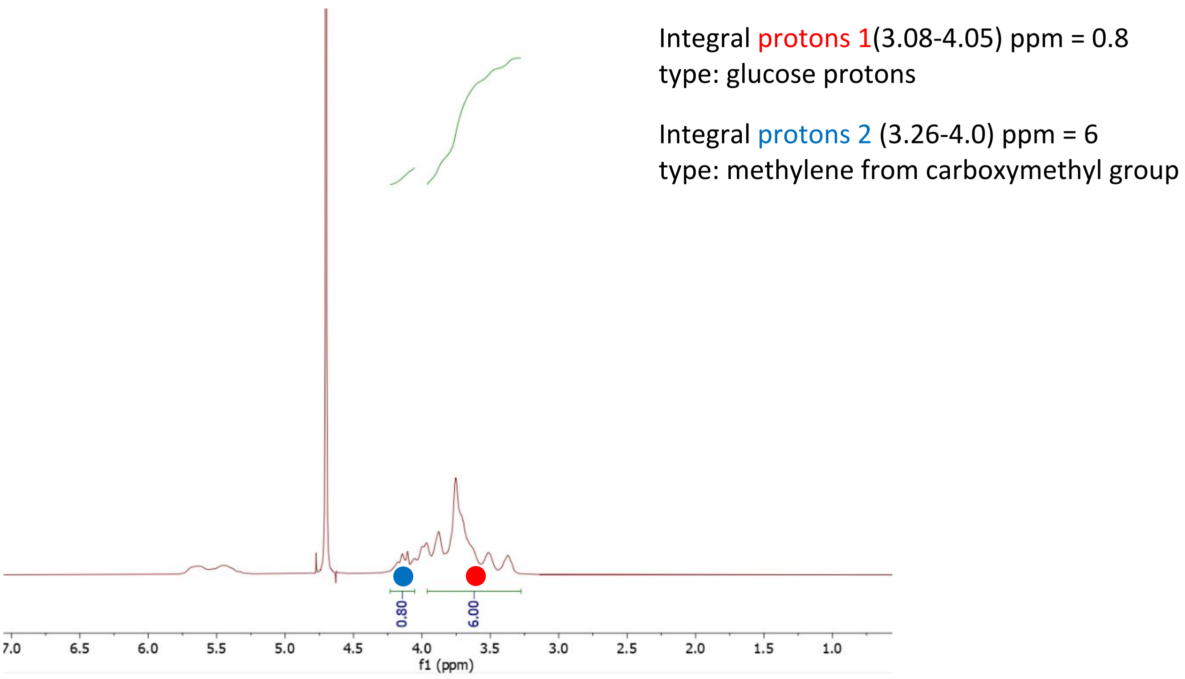

Figure S13. ^{1}H NMR of carboxymethyl-amylose.

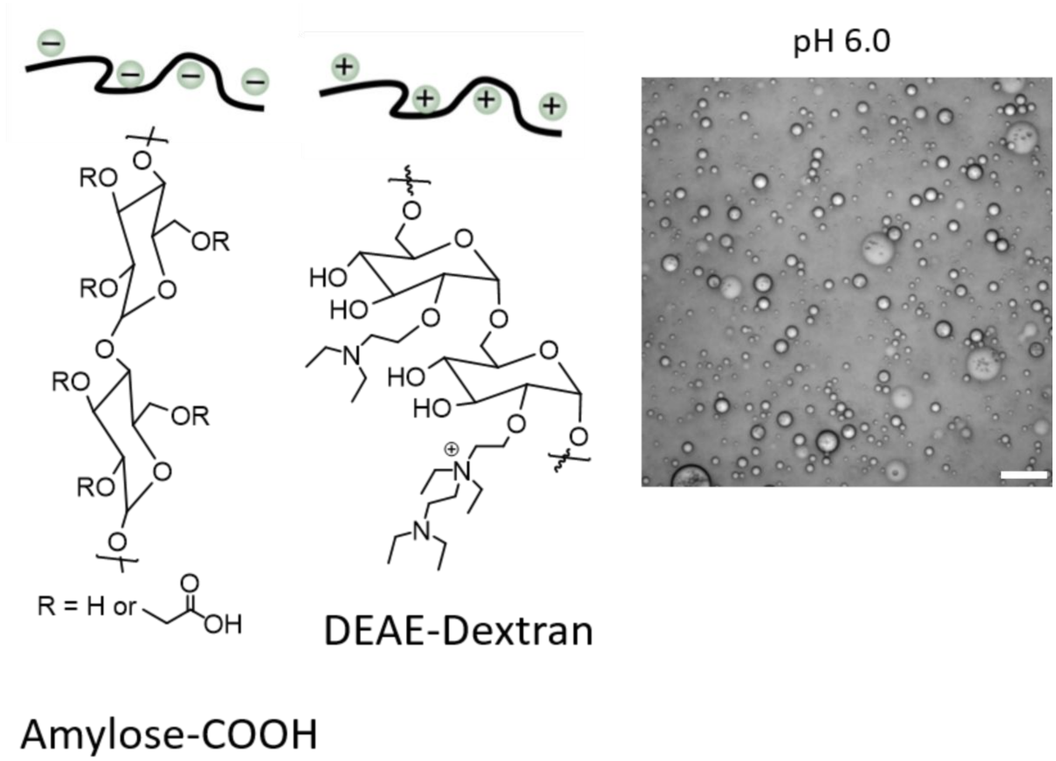

Figure S14. Chemical structure of coacervate-forming components. The micrograph shows the droplets resulting from the liquid-liquid phase separation observed when the components are mixed at pH 6. The concentration of the coacervate-forming components was 5 mg ml^{-1} for both DEAE-Dextran (Mw 10000 g mol^{-1}) and Amylose-COOH (Mw approximately 15000 g mol^{-1}) dissolved in 5 mM HEPES buffer. Bright-field imaging. Scale bar = 20 µm.

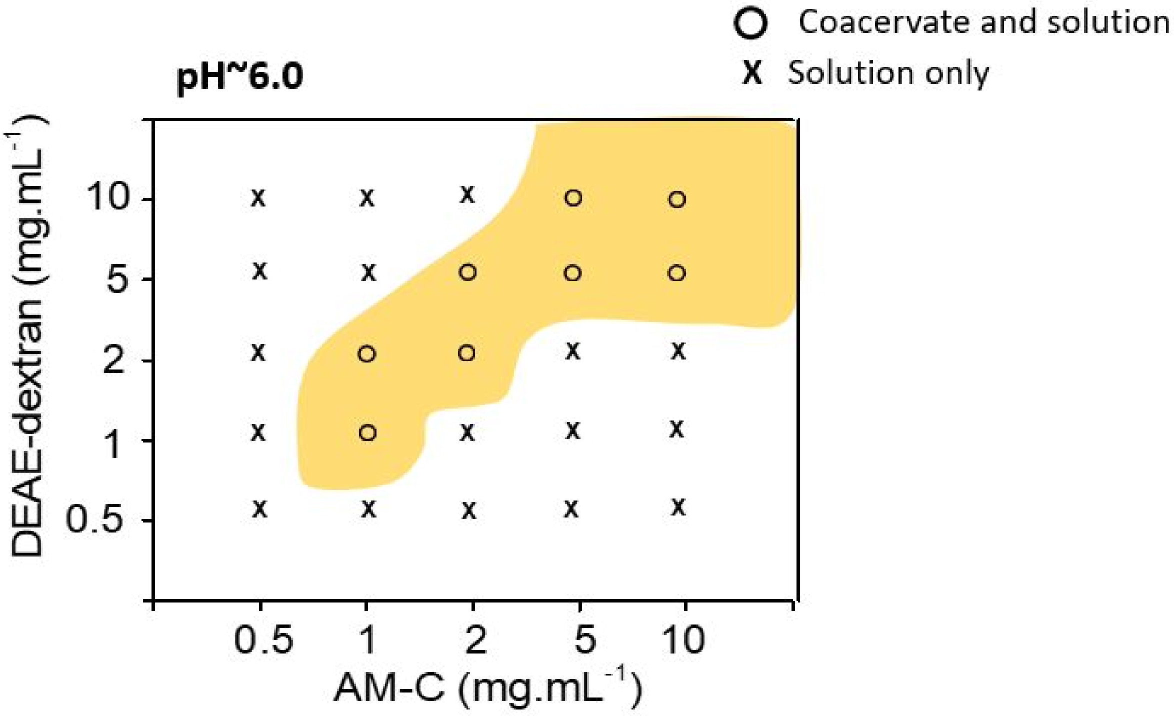

Figure S15. Phase diagram of the coacervation process. Obtained for different ratios between of Amylose-COOH and DEAE-Dextran. Coacervates form in the ranges of 1 to 10 mg $ml^{-1}$and if the ratio is approximately 1:1. Bright-field imagining.

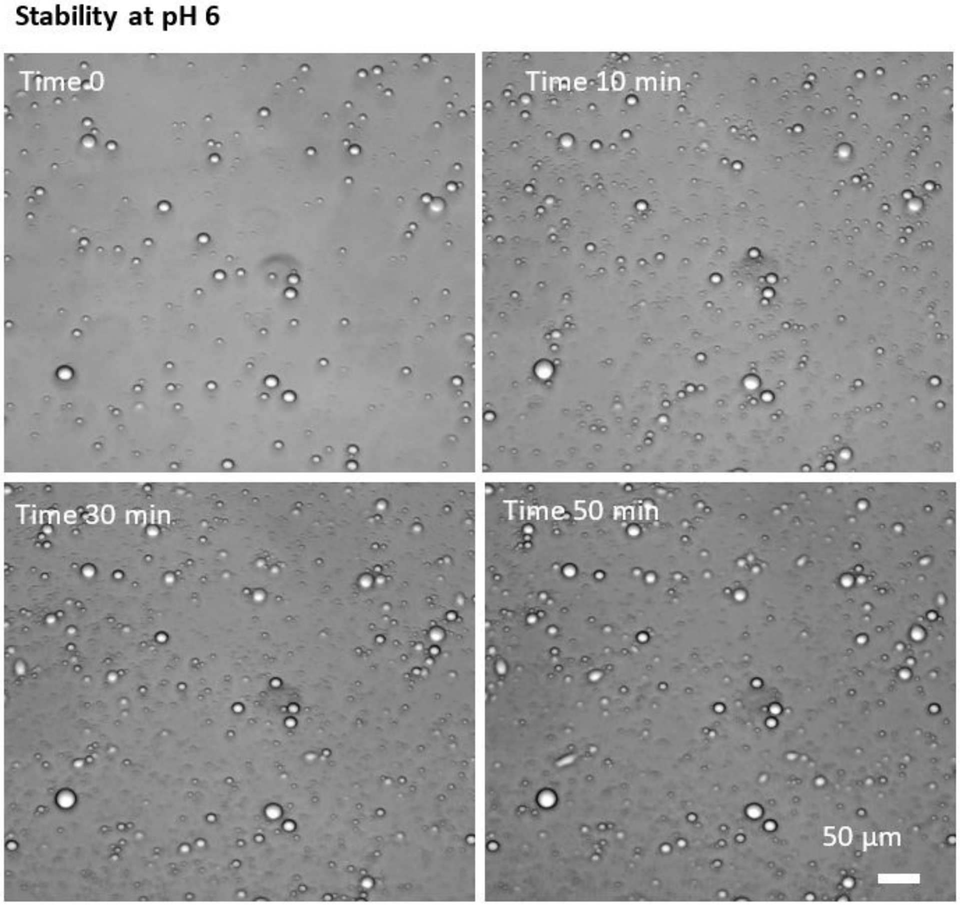

Figure S16. Stability of coacervates over 1 hour. The droplets are relatively stable over a period of 1 hour with components at a concentration of 2 mg ml^{-1} each. Few droplet fusion events were observed. The hydrodynamic diameter was calculated at each time point. At time 0 min the mean diameter was 13.0 µm with a standard deviation (SD) of 1.3; at 10 min the mean diameter was 13.4 µm with SD 0.8; at 30 min the mean diameter was 15.1 µm with SD 3.1; at 50 min the mean diameter was 16.1 with SD 2.6. Bright-field microscopy. The scale bar applies to all images.

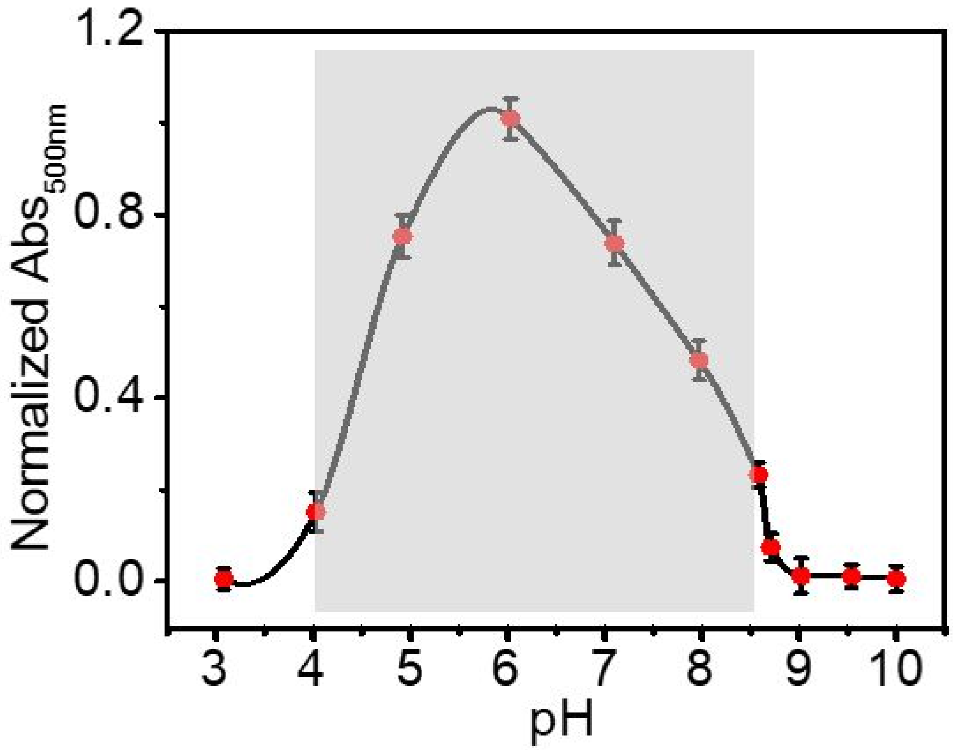

Figure S17. Formation of coacervates in buffer at different pH values. The concentration of amylose-COOH and DEAE-Dextran was 5 mg.ml^{-1} each. Coacervation was observed between pH values of 4 and 8.5. Optimal value around pH 6. Highly acidic or basic pH environments are unfavourable for droplet formation. Data based on the scattering behaviour of the dispersion (turbidity). Turbidity was measured by UV-vis spectrophotometry.

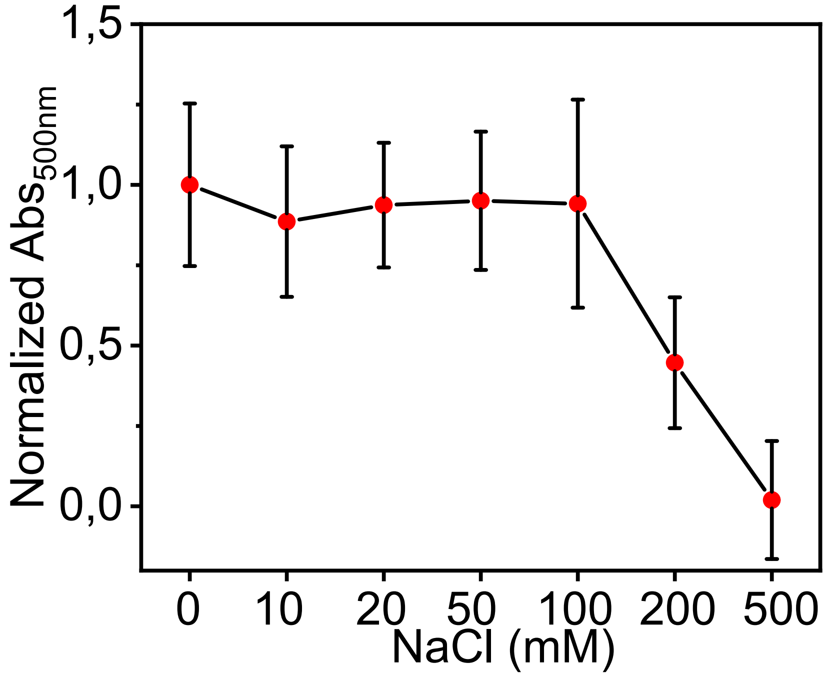

Figure S18. The resistance of coacervate droplets at different concentration of salts in the medium. The coacervates are relatively stable in NaCl concentrations up to 100 mM. Rapid disassembly of coacervate droplets was observed for concentrations above 200 mM. Data based on the scattering behaviour of the dispersion (turbidity). Turbidity was measured by plate reader.

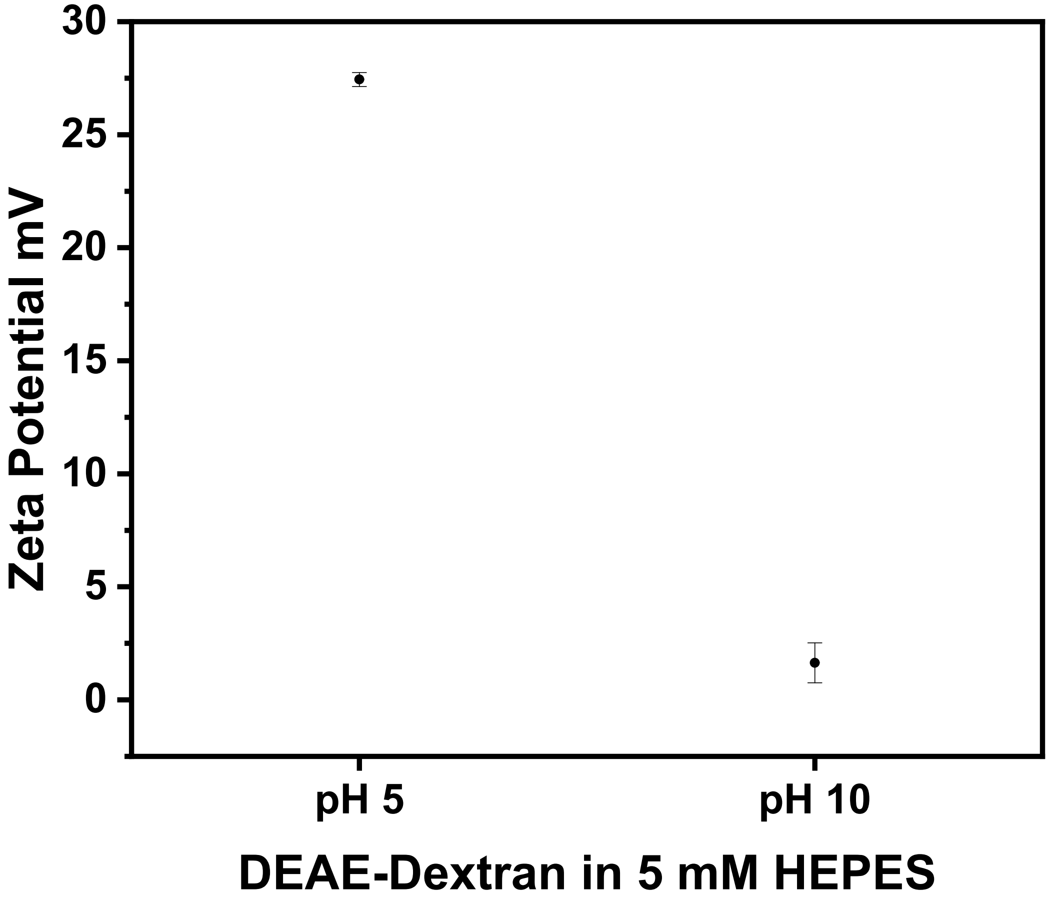

Figure S19. Zeta potential of a solutions containing DEAE-Dextran at pH values of 5 and 10. The concentration of DEAE-dextran was 2.5 mg ml^{-1} in 5 mM HEPES solutions at the corresponding values. The data was acquired by 3 runs of 5 measurements and the results showed that at pH 5 the DEAE-dextran has a charge of +27.44 ± 0.31 mV and at pH 10 it has a charge of +1.64 ± 0.88 mV.

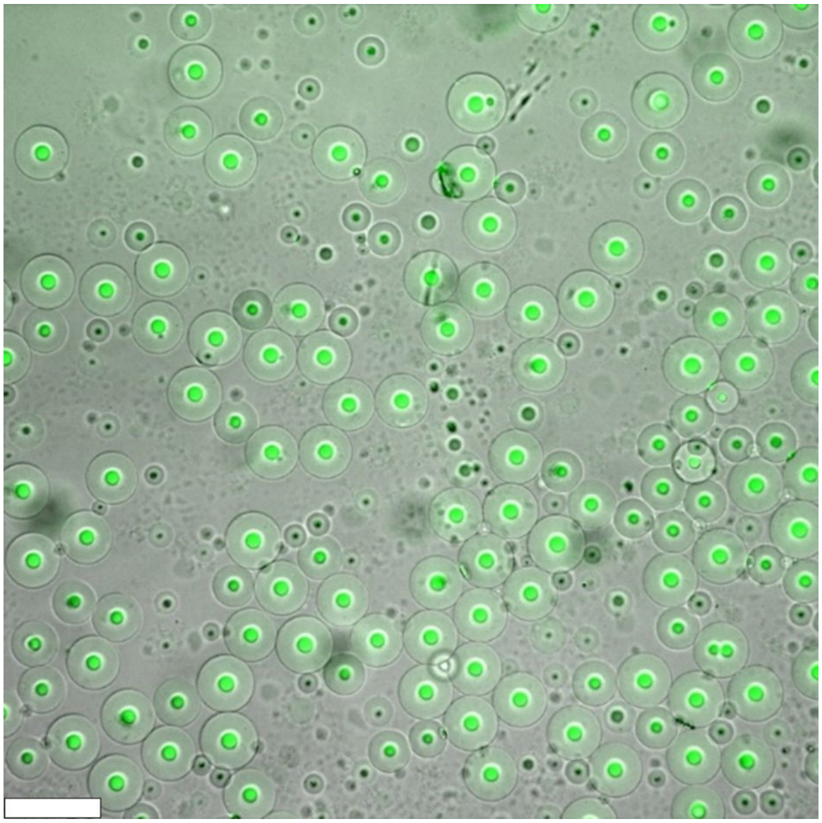

Figure S20. Coacervation in pGUVs. The image was taken 30 minutes after pH change from 11 to 5. All vesicles in the solution showed internal coacervation. DEAE-Dextran was tagged with FITC dye for visualization. The micrograph shows bright field and fluorescence channels combined. Fluorescence microscopy. Scale bar: 100 µm.

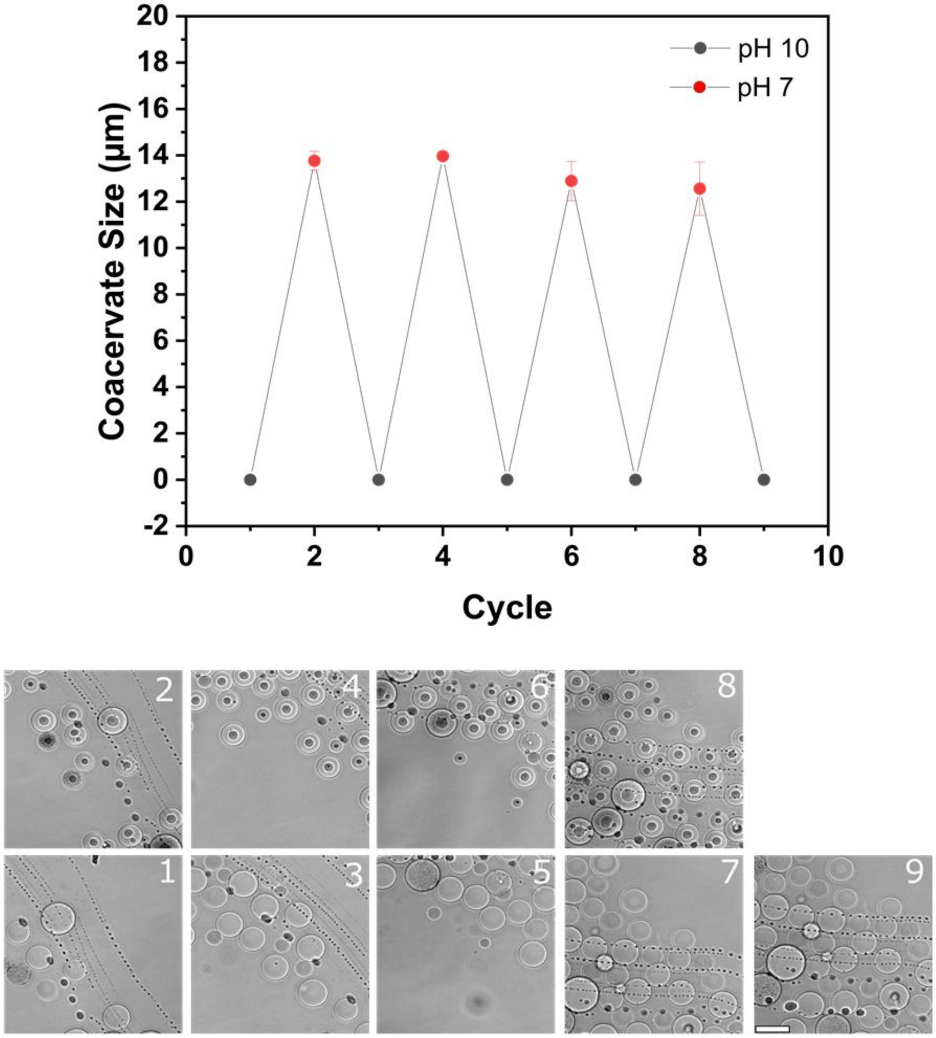

Figure S21. Cyclic dynamic formation of coacervates in pGUVs. Vesicles containing 5 mg mL^{-1} of AMC and DEAE were subjected to multiple cycles of pH step changes from 7 to 10. Coacervate droplet size data represent the average of five droplets measured from independent vesicles as

observed in bright field microscopy images. The results showed no coacervate formation at pH 10 and coacervates with diameters of approximately 13-14 µm at pH 7. Scale bar = 50 µm.

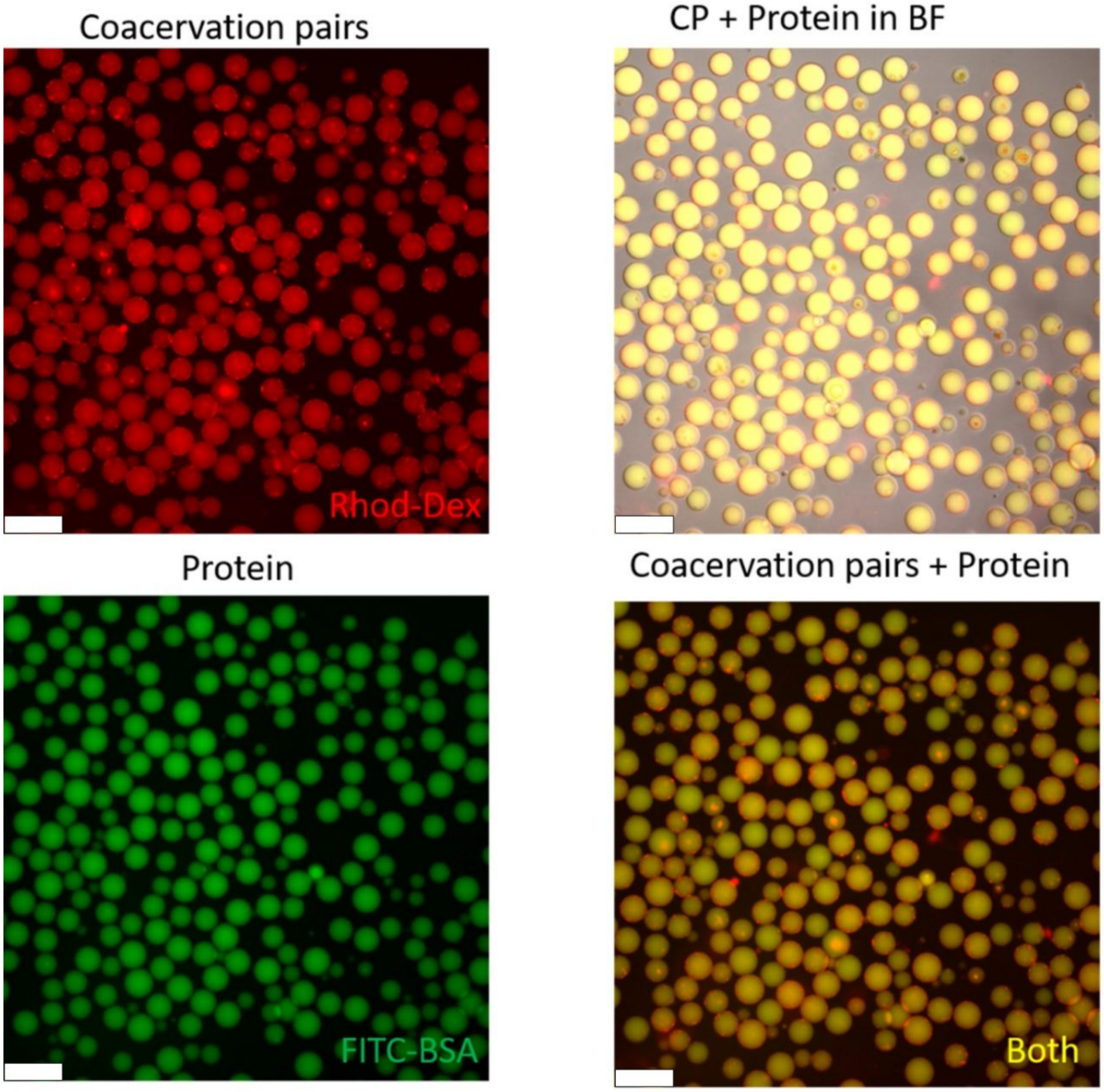

Figure S22. Vesicles containing a labelled protein (FITC-BSA) and the components needed for coacervation (DEAE-Dextran, Amylose-COOH). Dextran was labelled with Rhodamine. Before

coacervation, all encapsulated components are homogenously distributed inside the vesicles. Fluorescence microscopy. Scale bars: 100 µm.

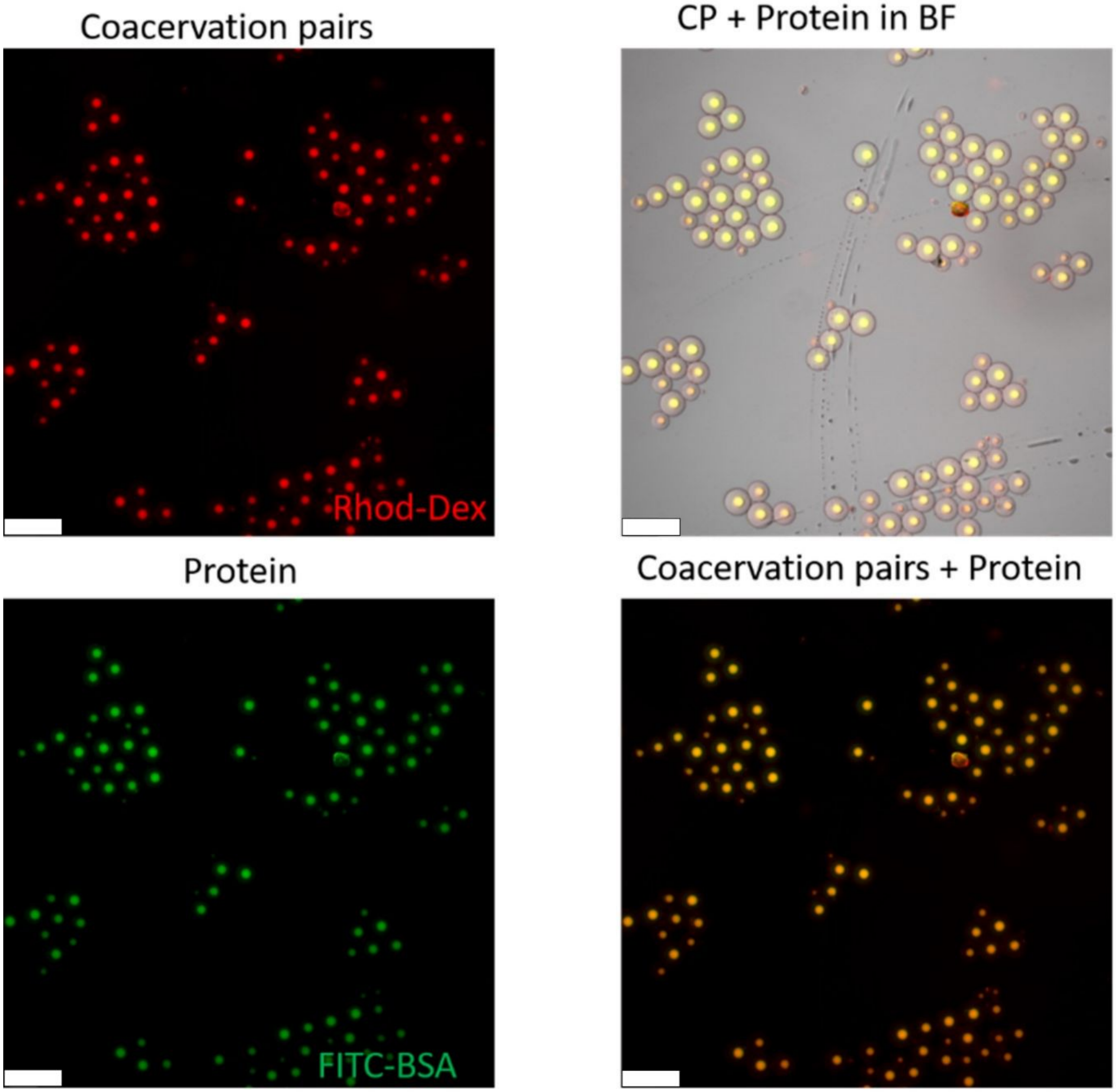

Figure S23. Vesicles containing a labelled protein (FITC-BSA) and the components needed for coacervation (DEAE-Dextran, Amylose-COOH). Dextran is labelled with Rhodamine. After coacervation, all components are colocalized within the coacervate droplets inside the vesicles. Fluorescence microscopy. Scale bars: 100 μm.

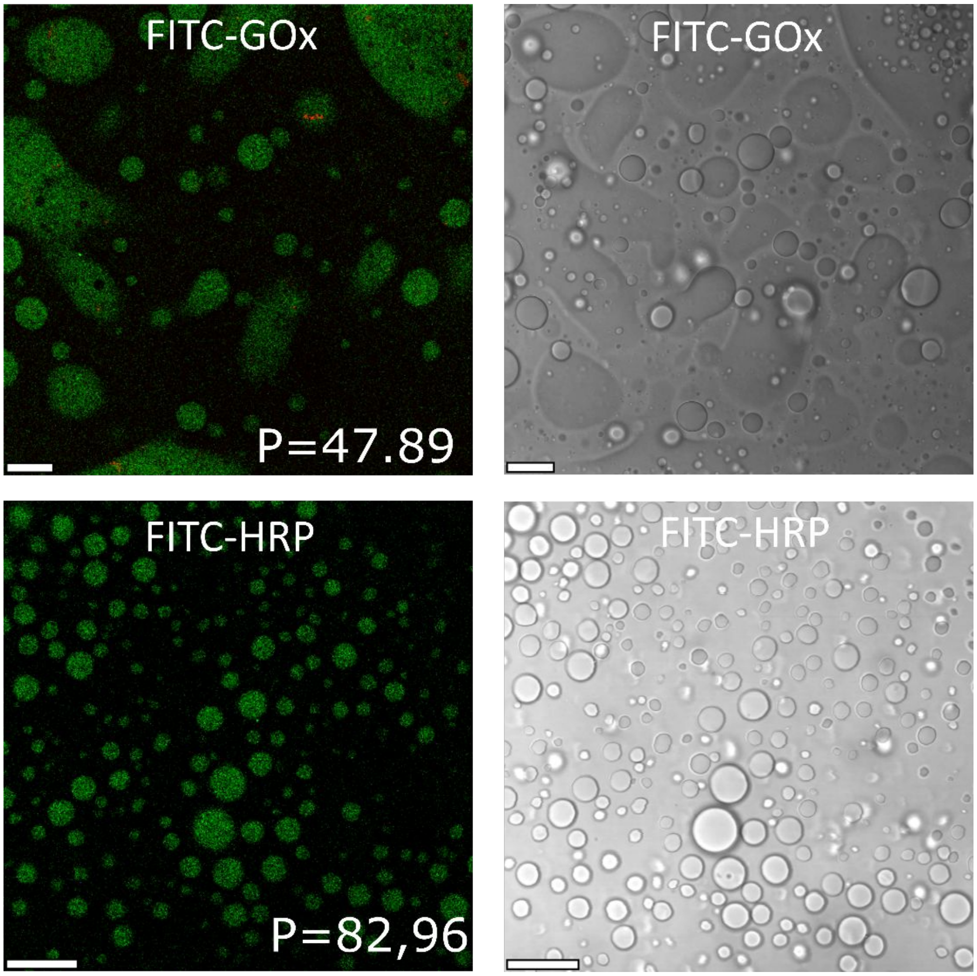

Figure S24. FITC-labelled HRP and GOx and their incorporation in coacervates. To prove encapsulation efficiency, the partitioning coefficient of the enzymes was determined using fluorescent CLSM data, which was analysed using ImageJ. The calculation was based on the average value of 5 randomly chosen points from within coacervates droplets containing the

enzymes, and points from the outer aqueous medium. The partition coefficient of FITC-HRP was 83, while the partition coefficient for FITC-GOx was 48. Scale bar = 20 μm.

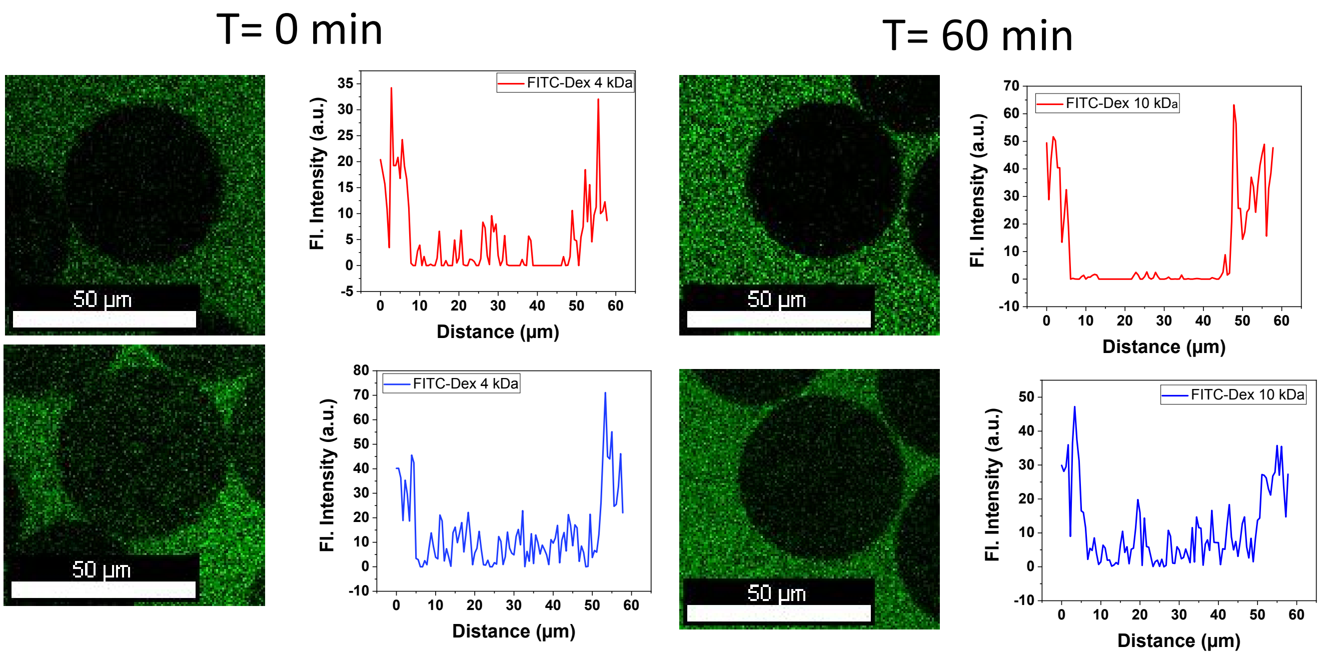

Figure S25. Large molecules cannot permeate the membrane. Permeability of pGUVs to larger molecules >1 kDa over a period of 1 hour. FITC-Dextran with molecular weight of 4 kDa (left) and 10 kDa (right). Confocal laser scanning microscopy.

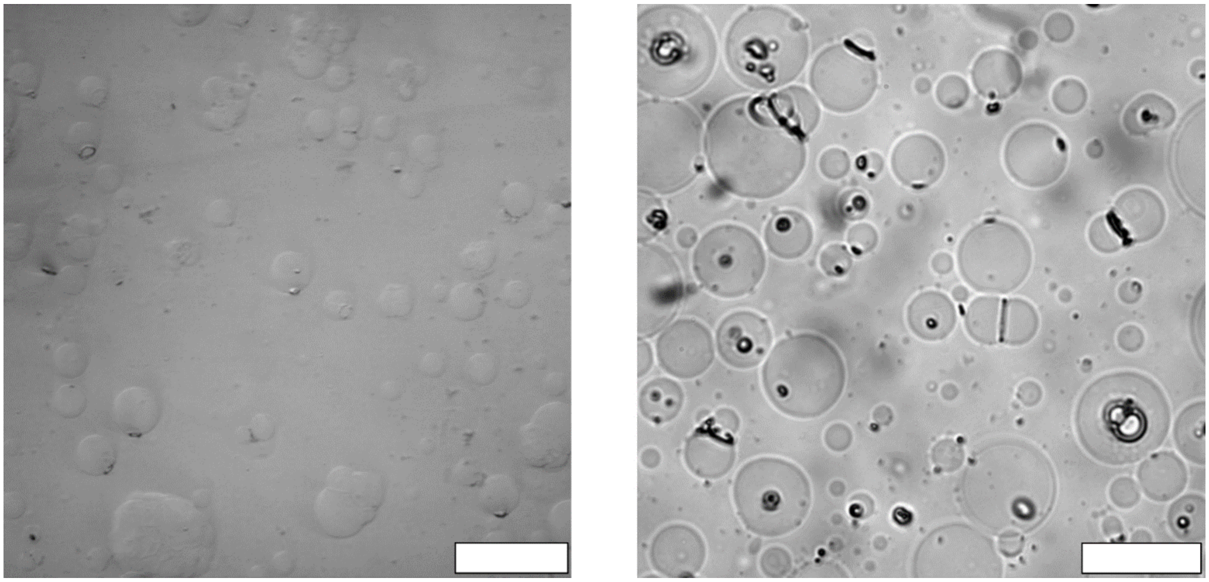

Figure S26. Formation of PB_{22}-PEO_{14} polymersomes by the double emulsion droplet formation method.[3] Polymer concentration is 10 mg ml^{-1} in toluene. Formation of these polymersomes was needed to compare how the different organic phases affect the permeability of the polymersomes.

Due to the fact that toluene was not compatible with PDMS-based microfluidics, the method for production of GUVs was double emulsion droplets (DED). The same concentrations of the components were used for both the microfluidics and DED methods with different organic phases, oleyl alcohol and toluene respectively. The resulted polymersomes had a high yield and polydispersity index and size comparable to the ones from microfluidics. Bright-field imaging. Scale bars 100 µm (left) and 60 µm (right).

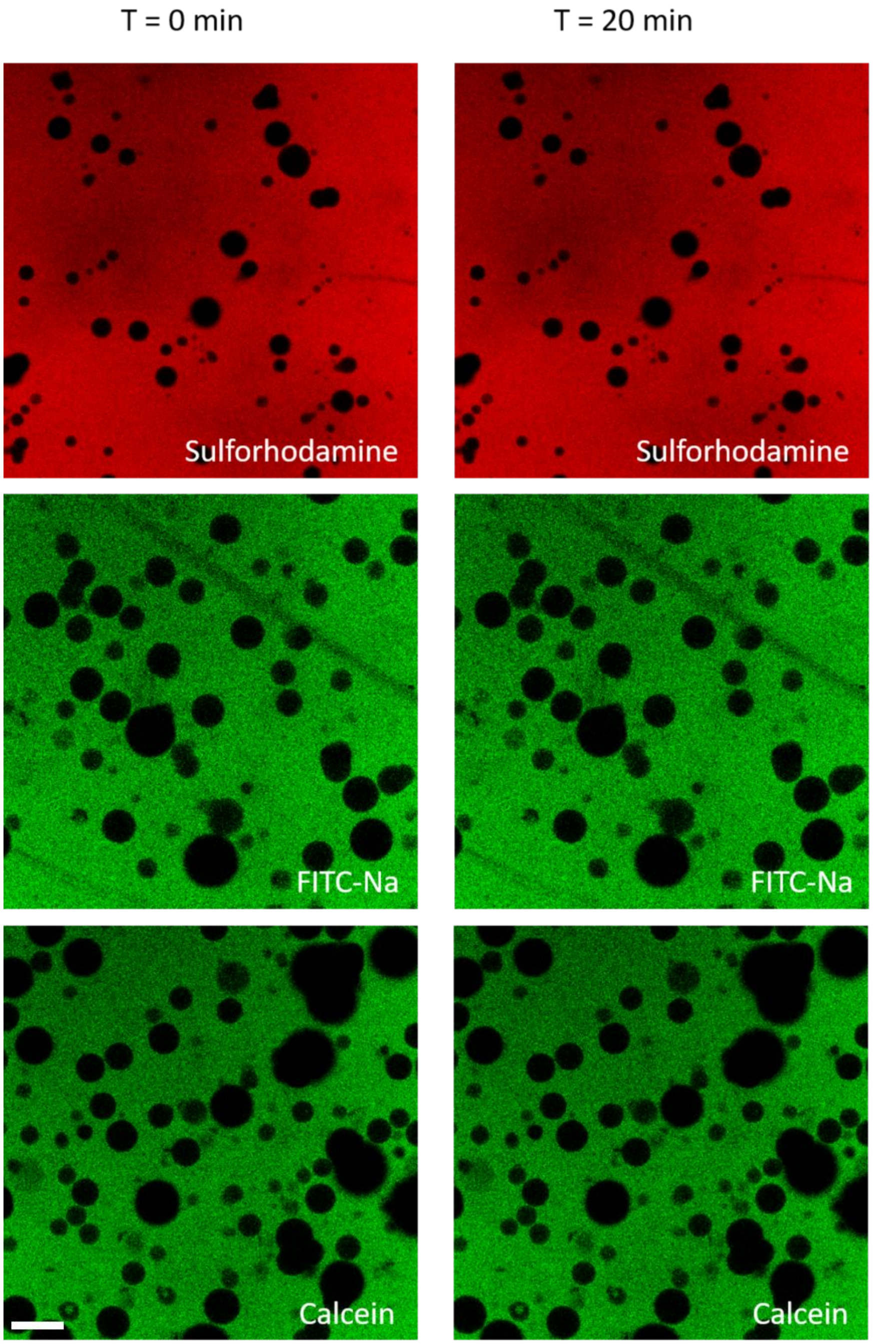

Figure S27. Permeability of polymersomes prepared by the oleyl alcohol-free method DED. The results showed no change in emission in their internal volume over a period of from 20 minutes to over 1 hour. The experimental concentration were the same as Figure 4a. The DED polymersomes

showed no permeability compared to their microfluidics counterparts. Confocal laser-scanning microscopy. Scale bar (applies to all images) = 50 µm.

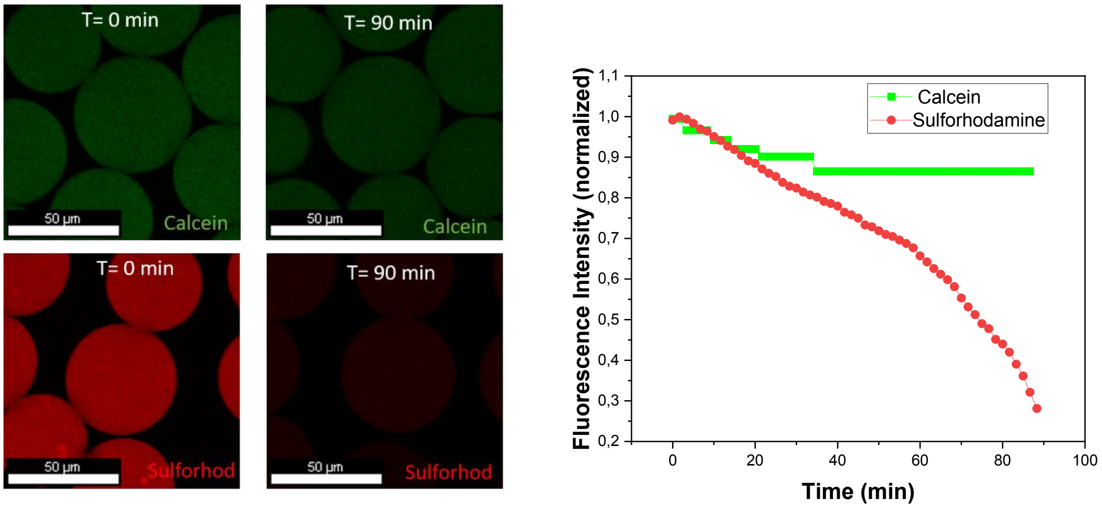

Figure S28. Dyes with different molecular weights were encapsulated in the polymersomes and their retention was studied. The dye with higher molecular weight calcein was successfully retained for more than 2 hours, while the sulforhodamine was able to diffuse in less than 1.5 hours. Confocal laser scanning microscopy. Scale bars: 50 µm.

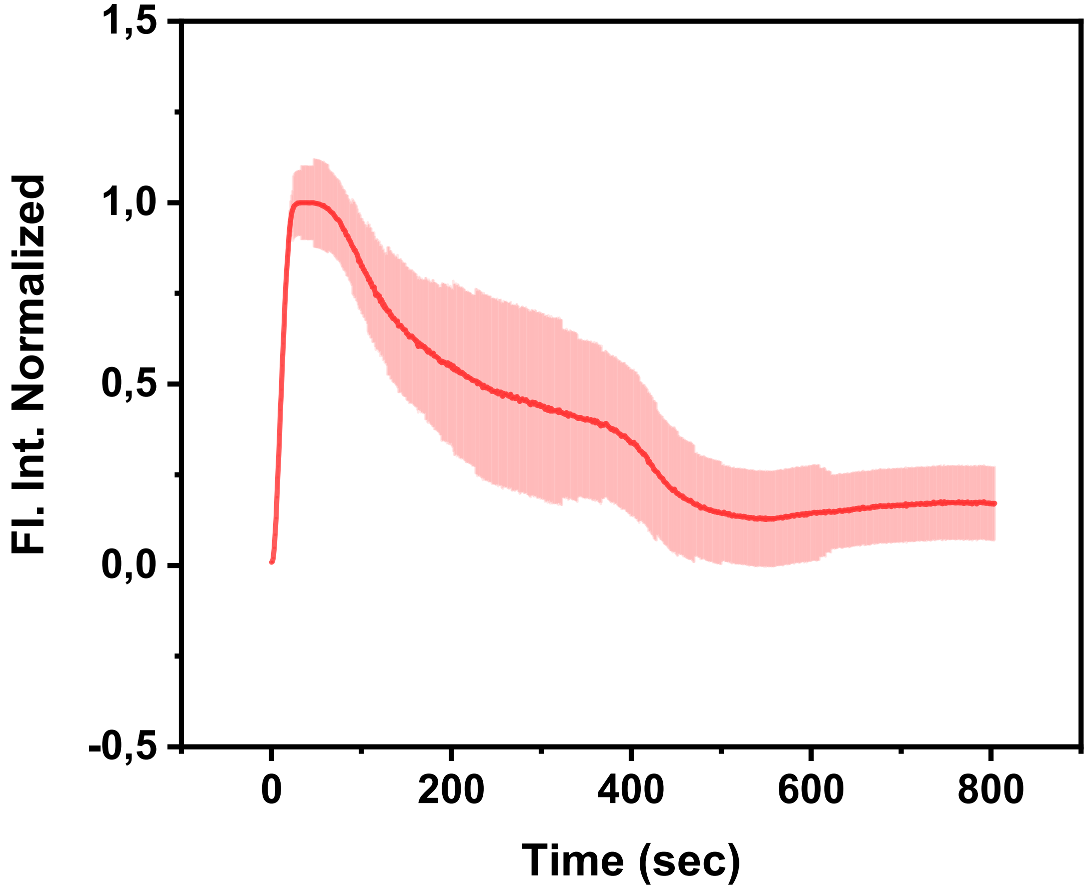

Figure S29. Kinetics of resorufin production in polymersomes between different batches. The concentration of the enzymes, HRP and GOx, within the polymersomes was 8 mg ml^{-1} and 50 μg ml^{-1}, respectively. The substrates, glucose (100 mM) and Amplex Red (25 μm mL^{-1}), were added externally. Data were acquired by laser scanning confocal microscopy at 1-second intervals. The data shown are the mean of a population of 30 polymersomes from 3 batches (10 polymersomes each) plotted using Origin software.

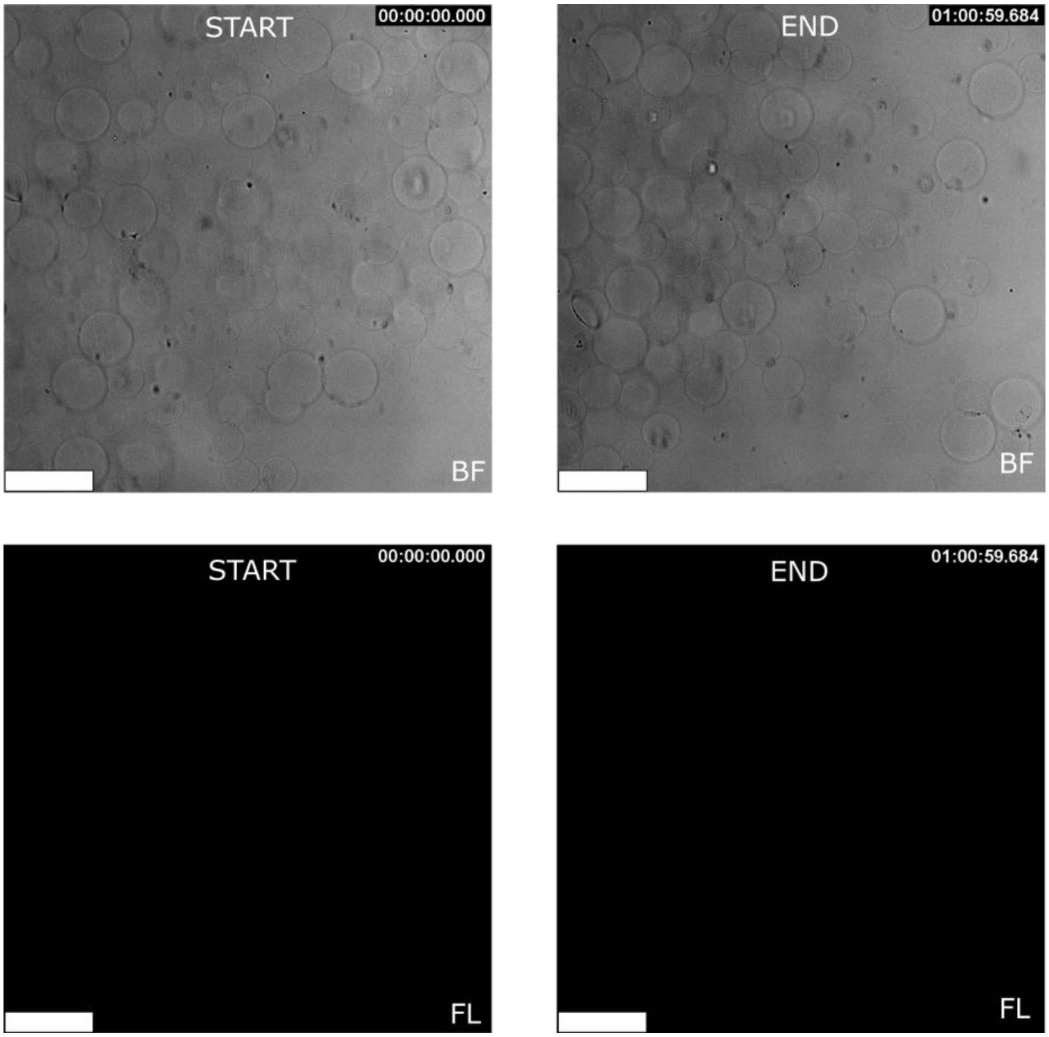

Figure S30. Control experiment for the diffusion of glucose across the polymeric membrane. To determine if the glucose indeed required around 1 hour to permeate the oleyl alcohol containing polymeric membrane, a control experiment was conducted without the 1-hour standby time. The results showed that production of resorufin is not possible without waiting for Glucose to permeate

the polymeric membrane for around 1 hour and indeed the membrane is intact and not easy to permeate. Scale bars = 100 μm. Confocal laser scanning microscopy.

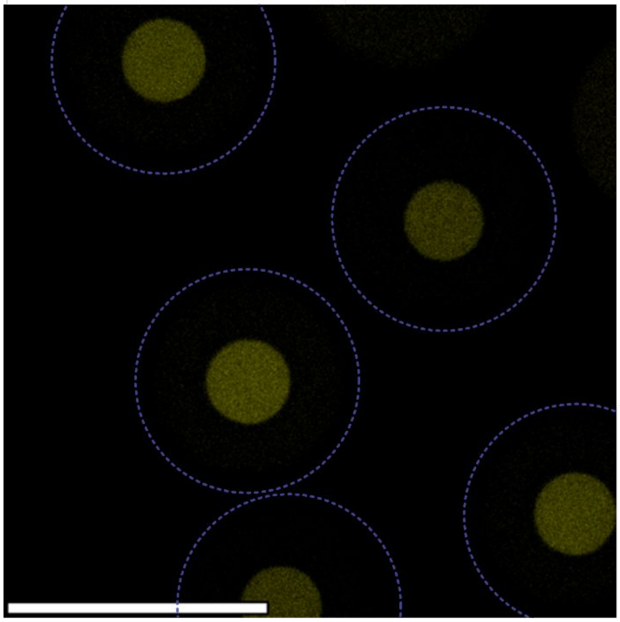

Figure S31. Production of resorufin in enzymatic microreactor. The DEAE-dextran and amylose-COOH were encapsulated in polymersomes at 5 $mg.ml^{-1}$ and HRP and GOx at 50 and 10 $\mu g.ml^{-1}$, respectively, at pH 11. The pH was lowered to 6 to induce LLPS and the formation of coacervates. Substrates for the enzymes were added externally to produce resorufin. The blue-dotted line indicates the polymer membrane. Confocal laser scanning microscopy, Scale bar = 50 μm.

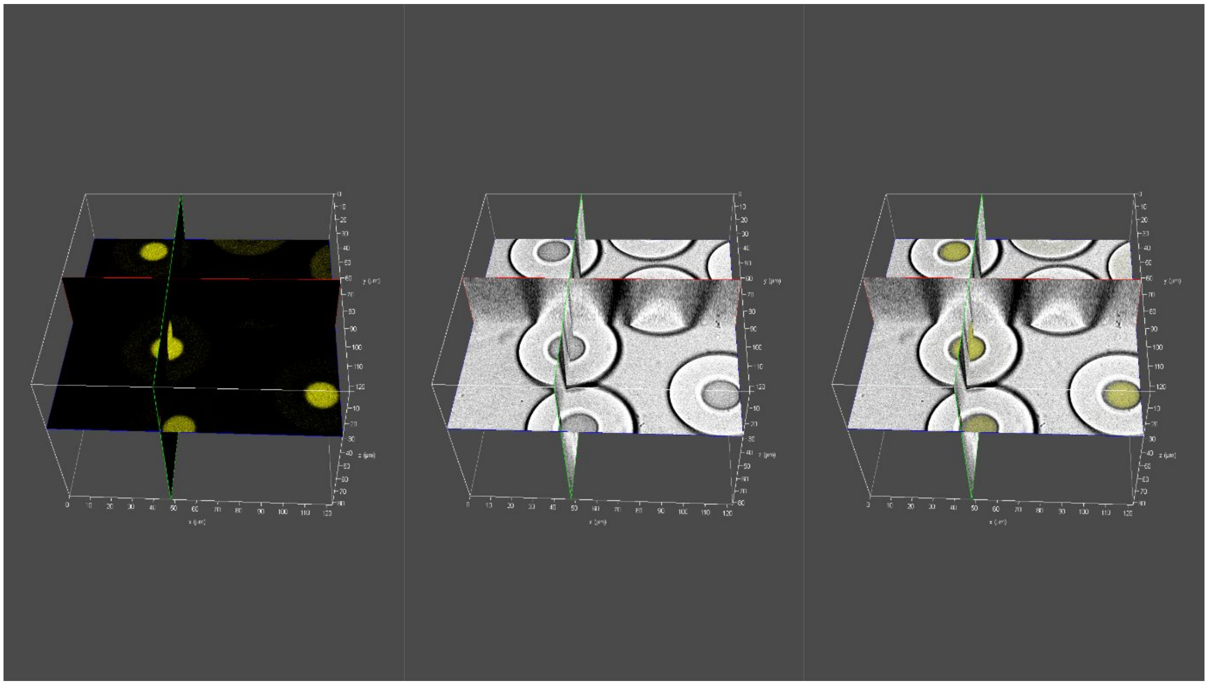

Figure S32. 3D images of the fluorescence channel, bright-field channel, and production of resorufin in coacervates in polymersomes. Data obtained by confocal laser scanning microscopy. Image was reconstructed using the Leica LASX software.
